# Metabolic and Inflammatory Risk Stratification in Resected Cholangiocarcinoma: A Retrospective Exploratory Analysis

**DOI:** 10.3390/medsci14050525

**Published:** 2026-08-28

**Authors:** Piya Prajumwongs, Attapol Titapun, Vasin Thanasukarn, Apiwat Jareanrat, Natcha Khuntikeo, Nisana Namwat, Poramate Klanrit, Arporn Wangwiwatsin, Jarin Chindaprasirt, Prakasit Sa-Ngiamwibool, Nattha Muangritdech, Sittiruk Roytrakul, Watcharin Loilome

**Affiliations:** 1 Cholangiocarcinoma Research Institute, Khon Kaen University, Khon Kaen 40002, Thailand; prajumwongspiya@gmail.com (P.P.); vasith@kku.ac.th (V.T.); nisana@kku.ac.th (N.N.); jarich@kku.ac.th (J.C.); prakasa@kku.ac.th (P.S.-N.); nattha.m@kkumail.com (N.M.); 2 Department of Surgery, Faculty of Medicine, Khon Kaen University, Khon Kaen 40002, Thailand; 3 Systems Biosciences and Computational Medicine, Faculty of Medicine, Khon Kaen University, Khon Kaen 40002, Thailand; 4 Medical Oncology Unit, Department of Medicine, Faculty of Medicine, Khon Kaen University, Khon Kaen 40002, Thailand; 5 Department of Pathology, Faculty of Medicine, Khon Kaen University, Khon Kaen 40002, Thailand; 6 Proteomics Research Laboratory, Genome Institute, National Center for Genetic Engineering and Biotechnology, National Science and Technology Development Agency, Pathum Thani 12120, Thailand; sittiruk@biotec.or.th

**Keywords:** cholangiocarcinoma, metabolite-based risk score, neutrophil-to-lymphocyte ratio, recurrence, prognostic stratification

## Abstract

**Background:** Cholangiocarcinoma (CCA) frequently recurs after curative-intent resection. Metabolic alterations and systemic inflammation may contribute to aggressive disease, but their combined prognostic relevance remains unclear. **Methods:** We retrospectively reanalyzed 88 patients with resected CCA and documented recurrence, including 37 with early and 51 with late recurrence. A metabolite-based risk score was developed from recurrence-associated serum metabolites identified previously. Model development and internal validation used all 88 patients, whereas analyses involving preoperative neutrophil-to-lymphocyte ratio (NLR), clinicopathological variables, and survival outcomes included 85 patients with complete clinical data. Performance was assessed using repeated nested cross-validation, bootstrap validation, discrimination, calibration, and survival analyses. **Results:** The 10-metabolite risk score (MRS-10) showed moderate internally validated discrimination for distinguishing early from late recurrence, with a pooled patient-level out-of-fold AUC of 0.828 (95% CI, 0.725–0.911), with limited calibration. High MRS was associated with shorter disease-free survival (DFS) and overall survival (OS) (both *p* < 0.001) and remained independently associated with DFS events (HR, 7.92; 95% CI, 3.94–15.92) and mortality (HR, 4.05; 95% CI, 2.08–7.89). High MRS was also associated with elevated NLR (*p* < 0.001). The combined MRS–NLR framework improved prognostic discrimination and stratified patients into groups with stepwise differences in DFS and OS. Exploratory subgroup analyses showed broadly consistent adverse associations across selected clinicopathological strata. **Conclusions:** In this retrospective exploratory analysis, a metabolite-based risk score combined with preoperative NLR was associated with recurrence and survival outcomes in resected CCA. This metabolic–inflammatory framework may provide complementary prognostic information alongside conventional clinicopathological factors. However, the findings remain hypothesis-generating and require validation in larger independent cohorts before clinical application.

## 1. Introduction

Cholangiocarcinoma (CCA) is an aggressive malignancy arising from the epithelial lining of the biliary tract and is associated with poor clinical outcomes despite advances in surgical and systemic therapies. Anatomically, CCA is commonly classified into intrahepatic, perihilar, and distal subtypes [1]; however, the present cohort included patients with intrahepatic cholangiocarcinoma (iCCA) and perihilar cholangiocarcinoma (pCCA), which represent clinically important subgroups in CCA management [2]. These subtypes differ in anatomical origin, clinical presentation, surgical management, recurrence patterns, prognosis, and molecular profiles. iCCA arises from the intrahepatic bile ducts within the liver, whereas pCCA develops at the hepatic hilum and involves the perihilar bile ducts. At the molecular level, iCCA is more frequently associated with alterations such as IDH1/2 mutations and FGFR2 fusions, whereas perihilar and other extrahepatic CCA subtypes more commonly show alterations involving pathways such as KRAS, TP53, and ERBB2, although frequencies vary across cohorts and etiologic backgrounds [3,4,5]. These subtype-related differences highlight the importance of considering tumor location when evaluating prognostic biomarkers in resected CCA. Surgical resection combined with adjuvant chemotherapy remains the only potentially curative treatment; however, long-term survival remains limited because of the high incidence of postoperative recurrence. Even among patients undergoing curative-intent resection, recurrence is common and represents a major cause of treatment failure and cancer-related mortality [6]. The aggressive biological behavior of CCA, together with its tendency for early dissemination, underscores the need for improved prognostic stratification after surgery [1,7]. Importantly, recurrence in CCA is not a uniform clinical event but reflects heterogeneous tumor biology. Early recurrence is often associated with aggressive tumor characteristics and rapid disease progression, whereas late recurrence may reflect more indolent residual disease or occult micrometastases present at resection. This heterogeneity highlights the limitations of conventional clinicopathological factors alone in capturing the biological diversity underlying recurrence risk and survival outcomes in resected CCA [1,8].

Metabolic reprogramming is a hallmark of cancer and contributes to tumor growth, invasion, immune evasion, and therapeutic resistance [9,10]. In CCA, alterations in amino acid metabolism, lipid remodeling, and energy production pathways have been linked to tumor aggressiveness and recurrence. Our previous serum metabolomics analysis showed that patients with different recurrence patterns exhibited distinct metabolic signatures, particularly involving dysregulated amino acids, lipid-derived carnitines, and glycerophospholipids [11]. These changes may reflect increased bioenergetic demand, fatty acid utilization, and membrane lipid remodeling during cancer progression. At the pathway level, recurrence-associated metabolites were mainly related to perturbations in amino acid metabolism, fatty acid β-oxidation and carnitine-mediated lipid transport, and glycerophospholipid metabolism. Several of these pathways are also connected to inflammatory and immune-related processes, including immune cell activation, pro-inflammatory lipid mediator synthesis, and immunometabolic reprogramming [12,13]. Thus, metabolomic profiling may capture biological information related not only to tumor-intrinsic aggressiveness but also to the inflammatory tumor–host microenvironment.

Although metabolomic profiling provides biologically informative data, the clinical interpretation of high-dimensional metabolomic results remains challenging. Individual metabolites may have limited reproducibility when interpreted in isolation, whereas complex prediction models may lack interpretability for routine clinical translation. Risk score-based approaches offer a practical strategy to summarize multiple biologically relevant metabolites into a single composite index, thereby reducing model complexity while preserving prognostic information. Previous studies in esophageal squamous cell carcinoma, hepatobiliary malignancies, and gastrointestinal cancers have shown that metabolite-based risk scores (MRS) can provide prognostic stratification and may support individualized risk assessment after curative treatment [14,15,16].

In addition to tumor-intrinsic metabolic alterations, host systemic inflammatory status plays an important role in cancer progression and outcomes. Systemic inflammation can promote tumor growth through immune suppression, pro-tumorigenic cytokine signaling, and modulation of the tumor microenvironment [17]. Among inflammation-related biomarkers, the neutrophil-to-lymphocyte ratio (NLR) is a simple, inexpensive, and widely available marker reflecting the balance between pro-inflammatory activity and anti-tumor immune response. Elevated NLR has been associated with poorer disease-free and overall survival across several solid tumors, including hepatocellular carcinoma [18], pancreatic cancer [19], colorectal cancer [20], and cholangiocarcinoma [21,22]. In our previous clinical analysis, elevated preoperative NLR was independently associated with worse survival outcomes in patients with resected intrahepatic cholangiocarcinoma, supporting the prognostic relevance of host inflammatory status in CCA [21].

Despite the prognostic relevance of both metabolic alterations and systemic inflammatory markers, these biological dimensions have largely been evaluated separately. Tumor-related metabolic reprogramming may reflect intrinsic biological aggressiveness, whereas inflammatory markers such as NLR capture host-related immune and inflammatory responses that influence disease progression and postoperative outcomes. The lack of an integrated framework combining metabolite-based risk information with systemic inflammatory status represents an important gap in postoperative prognostic assessment for CCA. Integrating these complementary dimensions may provide a more comprehensive characterization of recurrence risk and survival outcomes than either approach alone.

**Figure 1:**
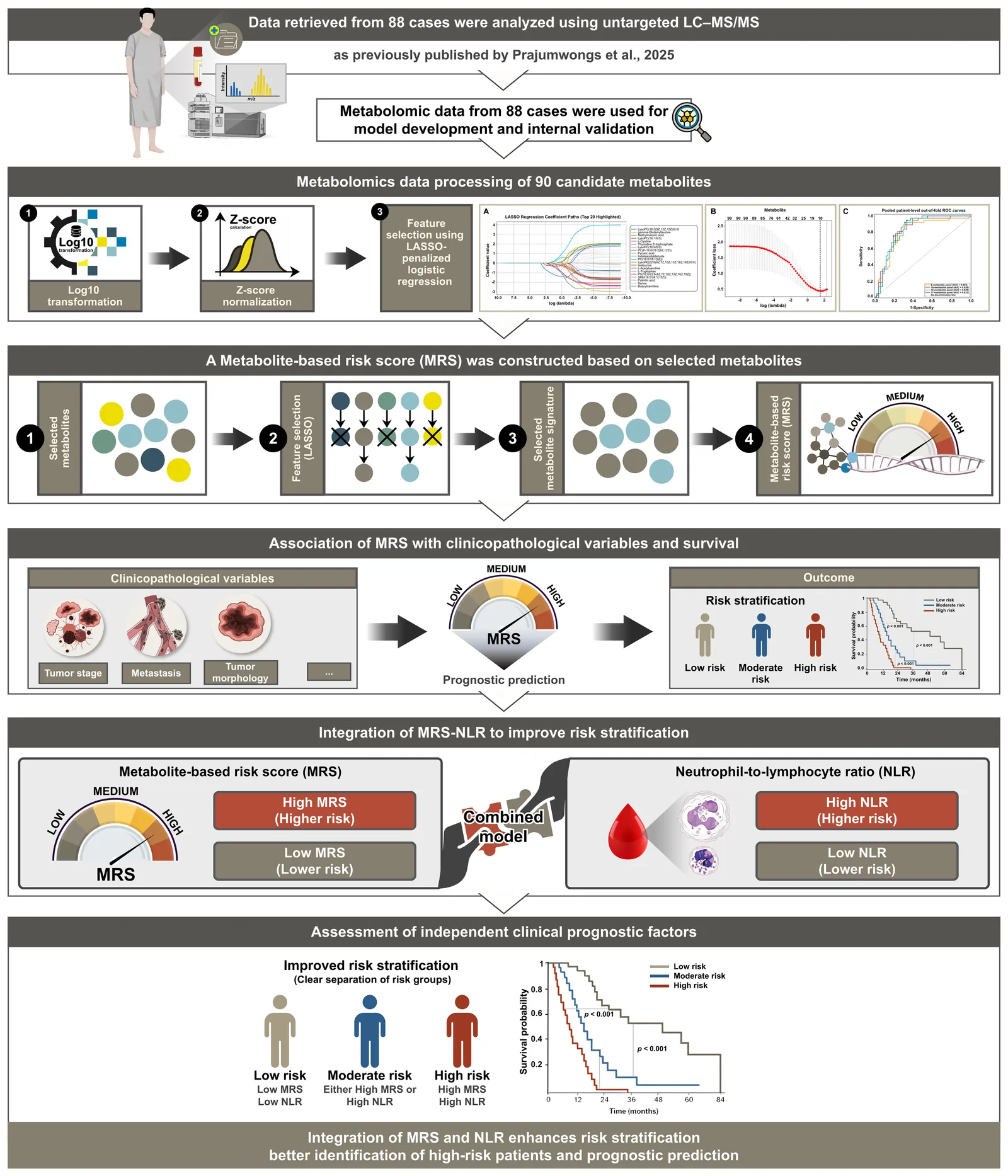
Analytical workflow of the study [11].

In this retrospective exploratory reanalysis of an existing cohort, we aimed to apply and evaluate a parsimonious MRS derived from previously identified recurrence-associated serum metabolites and to examine its association with disease-free and overall survival in patients with resected CCA. We further explored whether combining MRS with preoperative NLR could provide additional prognostic stratification and whether these associations were observed across selected clinically relevant subgroups, including tumor location and adjuvant chemotherapy exposure (Figure 1).

## 2. Materials and Methods

### 2.1. Study Design and Ethical Approval

This study was conducted as a retrospective secondary analysis of previously collected clinical and metabolomic data. The study protocol was reviewed and approved by the Khon Kaen University Ethics Committee for Human Research (reference number HE661318) and was conducted in accordance with the Declaration of Helsinki and Good Clinical Practice guidelines.

### 2.2. Study Population and Data Source

The study cohort was derived from our previously published serum metabolomics study of patients with CCA who underwent curative-intent surgical resection at Srinagarind Hospital, Faculty of Medicine, Khon Kaen University, Thailand, between 1 January 2017, and 31 December 2021 (Prajumwongs et al., 2025) [11]. The present study was a retrospective exploratory reanalysis of the 88 patients included in our previous untargeted LC–MS/MS metabolomics study. All patients had complete metabolomic data and documented recurrence timing and were therefore included in MRS model development and internal validation. Clinicopathological data, including demographic characteristics, tumor-related variables, pathological findings, and treatment information, were retrospectively retrieved from electronic medical records and the ISAN Cohort database maintained by the Cholangiocarcinoma Research Institute. Three patients lacked the preoperative white blood cell data required for NLR calculation; consequently, analyses involving NLR and the combined MRS–NLR framework were restricted to 85 complete cases. The study design, patient selection, and derivation of the model-development and complete-case clinical analysis cohorts are summarized in Figure 1.

### 2.3. Preoperative Neutrophil-to-Lymphocyte Ratio Assessment

Preoperative hematological data were retrieved from routine complete blood count tests obtained within one week before surgery, consistent with the approach previously reported by Chindaprasirt et al. [21]. If more than one preoperative complete blood count result was available during this period, the value closest to the date of surgery was used for NLR calculation. NLR was calculated as the ratio of absolute neutrophil count to absolute lymphocyte count. Patients were stratified into low and high NLR groups using an NLR cut-off value of 2.4, which corresponded to the median NLR value reported in the previously published cohort by Chindaprasirt et al. [21]. This approach was used to ensure consistency with prior evidence and to minimize data-driven threshold selection.

### 2.4. Metabolomic Data Source and Candidate Metabolite Selection

The metabolomic dataset was obtained from our previously published untargeted LC–MS/MS-based serum metabolomics analysis of patients with resected CCA [11]. The present metabolite-based model development cohort comprised the same 88 patients included in the previous metabolomic analysis, representing complete patient overlap between the two studies. Accordingly, the current cohort should not be considered an independent external validation cohort. In the previous study, recurrence-associated metabolites were identified by comparing metabolic profiles between patients with early and late recurrence. Metabolites meeting the combined criteria of a variable importance in projection (VIP) score > 1.2, a fold change (FC) > 1.2 or <0.83, and an FDR-adjusted *p*-value < 0.05 were retained after removal of duplicate annotations. Metabolites with lower discriminatory performance or analytically complex profiles were subsequently excluded, resulting in a predefined set of 90 candidate metabolites. These 90 metabolites were used as the candidate feature set for model development in this study.

Although the previous study used separate training and testing datasets for machine learning-based classification of recurrence patterns, that data partition was not retained in the current analysis because the modelling objectives differed. The previous study focused on metabolomics-based classification of early versus late recurrence, whereas the present study aimed to construct a parsimonious and clinically interpretable MRS, evaluate its associations with disease-free survival (DFS) and overall survival (OS), and assess its integration with preoperative NLR for postoperative risk stratification. All 88 patients had available metabolomic data and documented recurrence timing and were therefore included in development and internal validation. Three patients lacked the preoperative white blood cell data required for NLR calculation; consequently, analyses involving NLR and the combined MRS–NLR framework were restricted to 85 complete cases. No model parameters, regression coefficients, decision boundaries, classification models, or data partitions from the previous study were reused.

However, because the 90 candidate metabolites had been selected using early-versus-late recurrence outcomes from the same patients, the present analysis represents an outcome-informed secondary model-development study rather than an independent validation study. This prior outcome-informed feature selection may contribute to residual optimism in the estimated model performance and was therefore considered when interpreting the internal-validation results.

### 2.5. Data Preprocessing and Feature Selection

A total of 90 candidate metabolites measured across 88 serum samples were included in the model development pipeline. Raw metabolite intensity values were transformed using log10(x + 1) to reduce right-skewness and stabilize variance. Missing metabolite values were imputed using the median estimated from the corresponding training data. The transformed and imputed features were then standardized using Z-score normalization. Within all cross-validation and resampling procedures, imputation and standardization parameters were estimated using training data only and subsequently applied to the corresponding validation or test data.

For model development, the binary outcome was recurrence timing after surgical resection, classified as early recurrence within 12 months and late recurrence at or beyond 12 months. All patients included in the model-development cohort had documented recurrence; therefore, no patients censored without recurrence or patients who died without documented recurrence were included in the logistic regression analysis. Logistic regression was selected because the prespecified modelling objective was to classify patients according to the clinically defined 12-month recurrence threshold, rather than to model recurrence time as a continuous time-to-event outcome.

Primary feature selection was performed using L1-penalized logistic regression, also known as the least absolute shrinkage and selection operator (LASSO). Candidate regularization parameters were evaluated using stratified five-fold inner cross-validation across a predefined grid of C values, where C represents the inverse of the regularization strength. Model discrimination at each candidate value was assessed using the area under the receiver operating characteristic curve (ROC–AUC).

The final regularization parameter was selected using a two-stage, performance-constrained procedure. The one-standard-error value was used to define an acceptable performance-tolerance region rather than to select the sparsest admissible model. First, C values with a mean inner cross-validated AUC at or above one standard error below the maximum mean AUC were retained. Second, among these candidate values, the C yielding a mean number of non-zero metabolites closest to the target candidate-panel size of approximately 17 was selected, with ties favoring the smaller C. The highest mean inner-CV AUC was observed at C = 0.0858 (mean AUC, 0.8995; SD, 0.0614; SE, 0.0274). The selected regularization parameter was C = 0.3981, corresponding to λ = 1/C = 2.5119 and log10(λ) = 0.400. At this selected value, the mean inner-CV AUC was 0.8755 (SD, 0.0270; SE, 0.0121), and the mean number of non-zero metabolites across the inner-CV folds was 15.6.

After refitting the LASSO model on the full analytical dataset using C = 0.3981, 17 metabolites with non-zero coefficients were retained as candidate predictors. These metabolites were ranked according to the absolute magnitude of their LASSO coefficients, and the 10 highest-ranked metabolites were selected to define the parsimonious 10-metabolite panel. The final 10-metabolite prediction model was then refitted using logistic regression restricted to the selected metabolites, with a very weak L2 penalty (C = 10^6^) to approximate an unpenalized logistic model. Details of the preprocessing, regularization-parameter evaluation, and feature-selection procedure are provided in Supplementary Table S1.

### 2.6. Internal Validation of the 10-Metabolite Model

To evaluate potential optimism and overfitting, additional internal validation of the complete 10-metabolite modelling pipeline was performed using bootstrap resampling and repeated nested cross-validation. For bootstrap validation, 200 bootstrap resamples were generated. In each bootstrap sample, the complete modelling pipeline, including preprocessing, LASSO-based feature selection, 10-metabolite construction, and performance assessment, was repeated. Model performance was first evaluated within the bootstrap sample and then tested on the original dataset. Optimism was calculated as the difference between bootstrap-sample performance and test performance on the original dataset. The optimism-corrected AUC was calculated as the apparent AUC minus the mean bootstrap-estimated optimism.

Repeated nested cross-validation was also performed to assess internal model robustness while minimizing information leakage during performance estimation. The outer loop consisted of repeated stratified five-fold cross-validation repeated five times, resulting in 25 outer test folds. Within each outer training fold, the complete model development pipeline was repeated, including preprocessing, λ selection, LASSO-based feature selection, metabolite ranking, and final 10-metabolite model fitting. The held-out outer test fold was used only for performance evaluation and was not used during preprocessing, feature selection, regularization parameter selection, or model construction.

Model discrimination was assessed using ROC–AUC. Prediction error was assessed using the Brier score. Calibration was evaluated using calibration intercept and calibration slope, estimated by fitting a logistic calibration model with the logit of the predicted probability as the predictor. Calibration curves were also generated for visual assessment. The internal validation results are summarized in Supplementary Tables S2 and S3.

### 2.7. Construction of Metabolite-Based Risk Score Models

The 17 metabolites retained by the full-data LASSO model were ranked according to the absolute magnitude of their LASSO coefficients. Nested candidate panels comprising the top 17, 12, 10, and 8 metabolites were evaluated separately using the repeated nested cross-validation framework, with discrimination and prediction error assessed using ROC–AUC and the Brier score, respectively. Based on the balance among internally validated predictive performance, model parsimony, analytical burden, and potential feasibility for future targeted-assay development, the 10-metabolite model was selected as the primary MRS model for subsequent exploratory prognostic analyses.

After selection of the 10 metabolites, the final logistic regression model was refitted using these metabolites with a very weak L2 penalty (C = 10^6^) to approximate an unpenalized model. The final MRS-10 was calculated as a weighted linear predictor using the coefficients obtained from this refitted model. Specifically, the MRS-10 linear predictor was calculated as β0 + ΣβiZi, where β0 represents the model intercept, βi represents the final logistic regression coefficient for each metabolite, and Zi represents the standardized log-transformed metabolite intensity. Missing values were imputed using metabolite-specific medians before log10(x + 1) transformation and Z-score standardization. The full list of the selected metabolites, LASSO coefficients used for ranking, final logistic regression coefficients, model intercept, and MRS-10 equation is provided in Supplementary Table S4.

For descriptive and subsequent exploratory prognostic analyses, patients were classified into low- and high-MRS groups using an apparent cutoff selected by maximizing the Youden index in the development cohort. Because this cutoff was derived from the same cohort used for model development, it requires independent validation before clinical application.

### 2.8. Clinical Outcomes

The clinical outcomes were DFS and OS. DFS was defined as the time from surgical resection to the first documented recurrence or death from any cause, whichever occurred first. Patients who did not experience a DFS event were censored at the date of their last follow-up. OS was defined as the time from surgery to death from any cause, with surviving patients censored at the date of their last follow-up. Recurrence was determined based on histopathological confirmation, radiologic evidence from ultrasound, computed tomography, or magnetic resonance imaging, or clinical documentation in medical records.

### 2.9. Software and Statistical Analysis

Data preprocessing, feature selection, model construction, and model evaluation were performed using Python (version 3.13.5). L1-penalized logistic regression with cross-validation, bootstrap validation, and repeated nested cross-validation were implemented using scikit-learn. Model discrimination was evaluated using ROC curve analysis and AUC. Calibration was assessed using calibration curves, calibration intercept, calibration slope, and Brier score. Figures were generated using matplotlib.

Clinical and survival analyses were performed using IBM SPSS Statistics, version 27 (IBM Corp., Armonk, NY, USA). Continuous variables were summarized as medians with interquartile ranges and compared using the Mann–Whitney U test. Categorical variables were presented as frequencies and percentages and compared using the chi-square test or Fisher’s exact test. DFS and OS were estimated using the Kaplan–Meier method and compared using the log-rank test. Cox proportional hazards regression analyses were performed to identify variables associated with DFS and OS. For each outcome, variables with *p* < 0.20 in the corresponding univariate analysis were entered into multivariate Cox models. Hazard ratios (HRs) and 95% confidence intervals (CIs) were reported.

The proportional hazards (PH) assumption was assessed for variables included in the main multivariable Cox models using Schoenfeld residuals. Both variable-specific and global PH tests were evaluated, and a PH test *p*-value < 0.05 was considered suggestive of possible PH deviation. Ridge-penalized Cox regression with an L2 penalty was additionally performed as a sensitivity analysis using the same covariates included in each corresponding multivariable Cox model. For each model, standard Cox HRs with 95% CIs, Schoenfeld χ^2^ statistics, PH *p*-values, global PH test results, ridge-penalized HRs, and consistency of association direction between standard and ridge-penalized Cox models were recorded.

For integrated prognostic stratification, patients were categorized into three groups based on combined MRS and NLR status: low risk, defined as low MRS and low NLR; moderate risk, defined as discordant MRS and NLR status, including either high MRS with low NLR or low MRS with high NLR; and high risk, defined as high MRS and high NLR. Survival outcomes were compared using Kaplan–Meier analysis and Cox regression models. The prognostic performance of MRS alone, NLR alone, and the combined MRS–NLR model was compared using Harrell’s concordance index (C-index), Akaike information criterion (AIC), and global likelihood ratio tests from Cox proportional hazards models. Subgroup analyses were conducted according to tumor location and adjuvant chemotherapy exposure. All statistical tests were two-sided, and *p* < 0.05 was considered statistically significant.

## 3. Results

### 3.1. Development and Performance Evaluation of Metabolite-Based Risk Score Models

**Figure 2:**
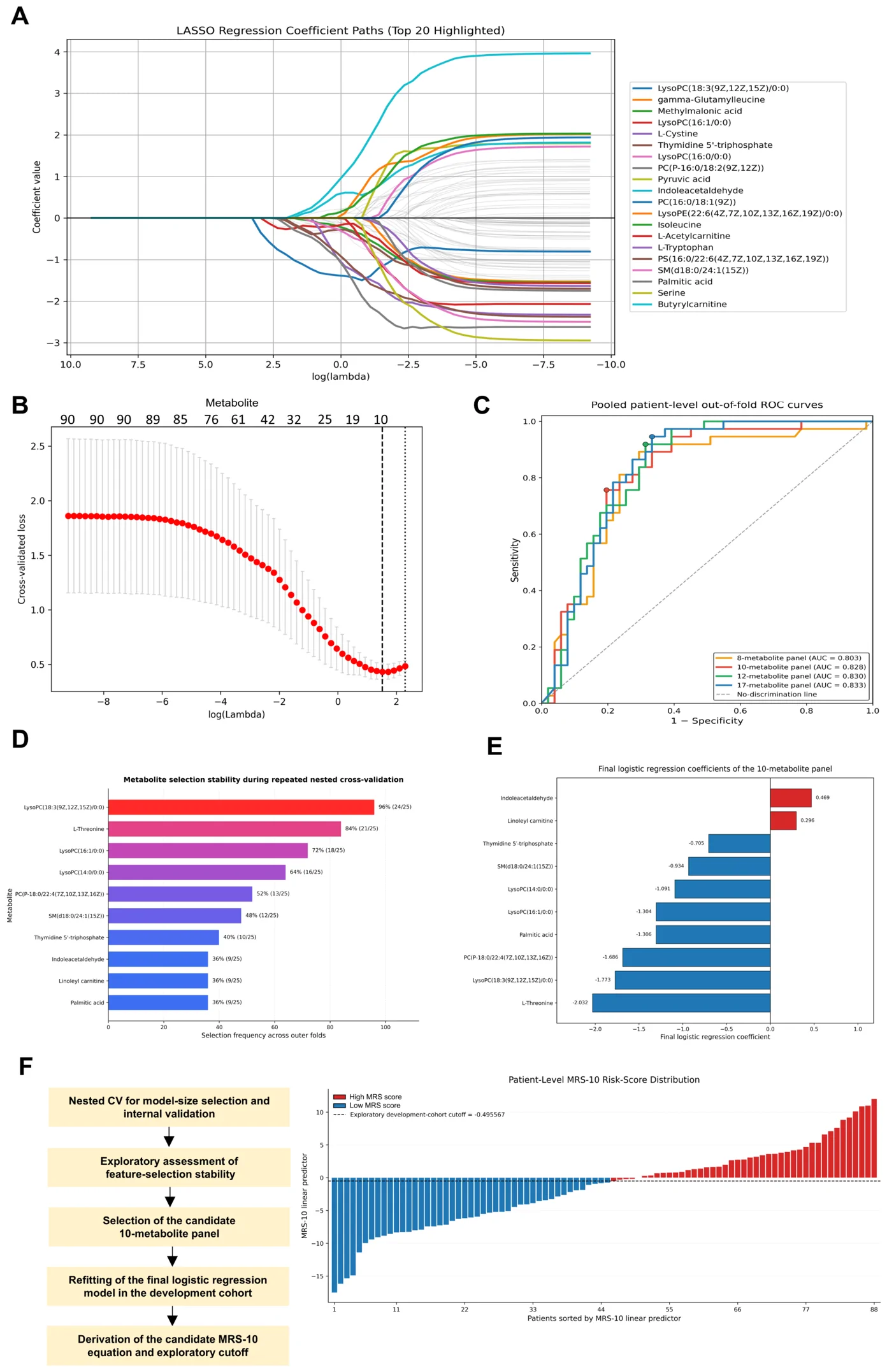
Development and internal validation of the candidate MRS-10 model. (**A**) LASSO coefficient paths for 90 candidate metabolites across the regularization path, with the 20 most prominent trajectories highlighted. (**B**) Stratified five-fold inner cross-validation profile used to select the regularization parameter. Red points show mean cross-validated loss, error bars show variability across folds, and numbers above the plot indicate retained metabolites. Dashed lines mark the candidate regularization parameters. (**C**) Pooled patient-level OOF ROC curves for the 17-, 12-, 10-, and 8-metabolite panels evaluated by repeated nested cross-validation. (**D**) Selection frequency of the final 10 metabolites across 25 outer-fold model-development iterations. (**E**) Final logistic regression coefficients after refitting the 10-metabolite model in the full development cohort. Positive coefficients indicate higher predicted probability of early recurrence. (**F**) Exploratory MRS-10 workflow and patient-level score distribution in 88 patients. Bars are ordered by the MRS-10 linear predictor, and the dashed line indicates the exploratory development-cohort Youden cutoff.

LASSO-based penalized logistic regression was applied to 90 candidate metabolites to identify features associated with recurrence timing. Stronger regularization, corresponding to smaller values of *C*, progressively shrank the regression coefficients toward zero and reduced model complexity, as illustrated by the coefficient trajectories in Figure 2A. Candidate regularization parameters were evaluated using stratified five-fold inner cross-validation. The highest mean inner-CV AUC was observed at *C* = 0.0858 (mean AUC, 0.8995; SD, 0.0614; SE, 0.0274), corresponding to an average of 1.8 non-zero coefficients.

The final regularization parameter was selected using a two-stage, performance-constrained procedure. The one-standard-error value was used to define an acceptable performance-tolerance region rather than to select the sparsest admissible model. Accordingly, candidate *C* values with a mean inner-CV AUC at or above one standard error below the maximum mean AUC were retained, yielding a lower performance boundary of 0.8721. Among these values, the *C* yielding a mean number of non-zero metabolites closest to the target candidate-panel size of approximately 17 was selected, with ties favoring the smaller *C*. This procedure selected *C* = 0.3981, corresponding to λ = 1/*C* = 2.5119 and log10(λ) = 0.400. At this selected parameter, the mean inner-CV AUC was 0.8755 (SD, 0.0270; SE, 0.0121), and the mean number of non-zero metabolites across the inner-CV folds was 15.6 (Figure 2B). The next less-regularized candidate, *C* = 0.5412, retained an average of 18.4 metabolites but had a mean inner-CV AUC of 0.8680, which fell below the predefined one-standard-error performance boundary. After refitting the LASSO model on the full analytical dataset using *C* = 0.3981, 17 metabolites retained non-zero coefficients (Supplementary Table S1).

The 17 retained metabolites were subsequently ranked according to the absolute magnitude of their LASSO coefficients. Nested candidate panels comprising the top 17, 12, 10, and 8 metabolites were evaluated separately using repeated nested cross-validation. The pooled patient-level OOF ROC curves are shown in Figure 2C. The 17-, 12-, 10-, and 8-metabolite panels yielded pooled OOF AUCs of 0.833 (95% CI, 0.730–0.903), 0.830 (95% CI, 0.727–0.904), 0.828 (95% CI, 0.725–0.911), and 0.803 (95% CI, 0.702–0.894), respectively. Their corresponding mean outer-fold AUCs were 0.796 (SD, 0.089), 0.817 (SD, 0.083), 0.819 (SD, 0.089), and 0.795 (SD, 0.080), respectively (Figure 2C and Table 1).

At the pooled OOF Youden cutoffs, the 10-metabolite panel achieved a sensitivity of 0.757, specificity of 0.804, and accuracy of 0.784. Although the 17-metabolite and 12-metabolite panels showed higher sensitivity, they had lower specificity than the 10-metabolite panel. Further reduction to eight metabolites resulted in lower pooled OOF and mean outer-fold AUCs. Because the confidence intervals of the 17-, 12- and 10- metabolite panels substantially overlapped, the 10-metabolite panel was not considered statistically superior. Rather, it provided the highest mean outer-fold AUC and the highest specificity while using fewer predictors than the 17- and 12-metabolite panels. The 10-metabolite panel was therefore selected as the parsimonious candidate model providing the most favorable balance among internally validated discrimination, model complexity, analytical burden, and potential feasibility for future targeted-assay development.

Feature-selection stability was assessed across the 25 outer-fold model-development iterations. A small core of metabolites, particularly LysoPC(18:3(9Z,12Z,15Z)/0:0), L-threonine, and LysoPC(16:1/0:0), was selected consistently, whereas several other metabolites showed lower selection frequencies, indicating incomplete stability in the exact composition of the broader panel (Figure 2D). After selection of the final 10 metabolites, the prediction model was refitted using logistic regression restricted to these features. The direction and magnitude of the final logistic regression coefficients are shown in Figure 2E, and the complete coefficient-based MRS-10 equation is provided in Supplementary Table S4.

Following comparison of the candidate 17-, 12-, 10-, and 8-metabolite panels using repeated nested cross-validation and assessment of feature-selection stability, the 10-metabolite panel was selected as the candidate parsimonious model. The panel was then refitted using logistic regression in the full development cohort, and the resulting coefficients and intercept were used to derive the MRS-10 linear-predictor equation. Patient-level MRS-10 scores and predicted probabilities were subsequently calculated for all 88 patients, comprising 37 patients with early recurrence and 51 patients with late recurrence, were included in the development-cohort analysis. When evaluated in the same cohort used for model development, the final refitted MRS-10 model achieved an apparent ROC–AUC of 0.973. An exploratory Youden-index probability cutoff of 0.378583, corresponding to a linear-predictor cutoff of −0.495567, was used to stratify patients according to their MRS-10 values. Based on this cutoff, 43 patients were classified into the high-MRS group and 45 patients into the low-MRS group. Figure 2F shows the patient-level distribution of the MRS-10 linear predictor, with patients ordered from the lowest to the highest score and bar colors indicating the observed recurrence group.

The high apparent AUC reflects evaluation of the final MRS-10 model in the same cohort used for feature selection, panel construction, and coefficient estimation. It therefore represents the model fit within the development dataset and is expected to be optimistic relative to performance in previously unseen patients. This apparent analysis was retained to define the final coefficient-based MRS-10 equation, illustrate the patient-level score distribution, and derive an exploratory development-cohort cutoff. Generalizable model performance was assessed separately using repeated nested cross-validation, which yielded a pooled patient-level out-of-fold AUC of 0.828 (95% CI, 0.725–0.911). Accordingly, the apparent AUC of 0.973 should be interpreted as development-cohort performance rather than as the primary internally validated estimate.

Clinical data required for the subsequent prognostic analyses were incomplete for three patients in low-MRS group. These patients were excluded from analyses involving clinical and survival variables, leaving 85 patients for the subsequent prognostic analyses.

### 3.2. Baseline Characteristics and Associations with MRS

After excluding three patients with incomplete clinical data, 85 patients were included in the subsequent clinicopathological analyses, comprising 42 patients in the low-MRS group and 43 patients in the high-MRS group. Clinicopathological characteristics of patients stratified by MRS are summarized in Table 2. Baseline demographic characteristics, including age and sex, were comparable between the low- and high-MRS groups, with no statistically significant differences observed. Tumor location also did not differ significantly between the two groups, with comparable proportions of intrahepatic and perihilar cholangiocarcinoma (*p* = 0.928).

With respect to surgical margin status, patients in the high-MRS group had a higher proportion of positive resection margins (R1) compared with those in the low-MRS group (53.5% vs. 31.0%), although this difference did not reach statistical significance (*p* = 0.060). Tumor morphology showed a significant association with MRS (*p* = 0.041). Specifically, the high-MRS group had a lower proportion of intraductal morphology (2.3% vs. 19.0%) and a higher proportion of mixed or non-intraductal growth patterns.

No significant difference in histological differentiation was observed between groups (*p* = 0.204), although poorly or moderately differentiated tumors tended to be more frequent in the high-MRS group. Regarding nodal status, patients with high MRS were significantly more likely to have lymph node involvement (N1) than those with low MRS (55.8% vs. 31.0%, *p* = 0.036). Advanced-stage disease (stage III–IV) was more common in the high-MRS group compared with the low-MRS group (86.0% vs. 69.0%), although this association did not reach statistical significance (*p* = 0.105).

MRS was significantly associated with systemic inflammatory status. High-MRS patients were had a higher proportion to exhibit elevated NLR compared with low-MRS patients (74.4% vs. 23.8%, *p* < 0.001). Chemotherapy exposure differed significantly between groups (*p* = 0.030), with patients in the low-MRS group more frequently receiving adjuvant chemotherapy, whereas a larger proportion of patients in the high-MRS group did not receive chemotherapy.

### 3.3. Univariate and Multivariate DFS and OS Analyses of MRS in Cholangiocarcinoma

Baseline demographic factors, including age and sex, were not significantly associated with either DFS or OS in univariate analyses and were therefore excluded from subsequent prognostic models.

For DFS, univariate analysis identified N1 disease, advanced stage, elevated NLR, and high MRS as significant adverse prognostic factors. Patients with N1 disease had shorter DFS than those with N0 disease (8.8 vs. 19.7 months, *p* = 0.012), while stage III–IV disease was associated with poorer DFS compared with stage 0–II disease (12.7 vs. 26.2 months, *p* = 0.045). Elevated NLR was significantly associated with reduced DFS (7.8 vs. 26.3 months, *p* < 0.001). Patients with high MRS showed shorter DFS than those with low MRS (7.4 vs. 32.0 months, *p* < 0.001). Tumor location, differentiation, tumor morphology, and resection margin status were not significant in univariate analysis.

For each outcome, variables with *p* < 0.20 in the corresponding univariate analysis were entered into the multivariate Cox proportional hazards model. In the DFS model, tumor location was independently associated with pCCA associated with a lower recurrence hazard than iCCA (HR 0.42, 95% CI 0.23–0.78, *p* = 0.006). High MRS (HR 7.92, 95% CI 3.94–15.92, *p* < 0.001) and elevated NLR (HR 2.84, 95% CI 1.55–5.20, *p* = 0.001) also remained independently associated with recurrence. Although N stage and TNM stage were significant in univariate analysis and were therefore included in the DFS multivariate model, they did not retain independent prognostic significance after adjustment.

For OS, univariate analysis showed that N1 disease, advanced stage, elevated NLR, no adjuvant chemotherapy exposure, and high MRS were significantly associated with shorter survival. Median OS was shorter in patients with N1 disease (15.9 vs. 25.9 months, *p* = 0.016), stage III–IV disease (16.9 vs. 38.5 months, *p* = 0.026), elevated NLR (12.7 vs. 32.1 months, *p* < 0.001), no adjuvant chemotherapy exposure (10.2 vs. 25.9 months, *p* < 0.001), and high MRS (10.7 vs. 38.5 months, *p* < 0.001). In the OS multivariate model, high NLR associated with higher mortality hazard than low NLR (HR 2.75, 95% 1.46–5.15, *p* = 0.002), while elevated adjuvant chemotherapy exposure (HR 0.32, 95% CI 0.18–0.59, *p* < 0.001), and high MRS (HR 4.05, 95% CI 2.08–7.89, *p* < 0.001) remained independently associated with OS. Although N stage and TNM stage were significant in univariate analysis and were therefore included in the OS multivariate model, they were no longer independently significant after adjustment (Table 3).

To evaluate the proportional hazards (PH) assumption and the stability of the main multivariable Cox estimates, PH assumption testing using Schoenfeld residuals and ridge-penalized Cox regression sensitivity analysis were performed. For the DFS model, Schoenfeld residual testing showed no evidence of global PH violation (global χ^2^ = 3.231, df = 7, *p* = 0.863). Ridge-penalized Cox regression showed directionally consistent associations for all included variables, including high MRS and elevated NLR (Supplementary Table S5). For the OS model, Schoenfeld residual testing also showed no evidence of global PH violation (global χ^2^ = 10.617, df = 8, *p* = 0.224). Ridge-penalized Cox regression similarly showed directionally consistent associations for the variables included in the multivariable model (Supplementary Table S6).

### 3.4. Risk Stratification for Recurrence and Survival Using Combined MRS and NLR

The prognostic performance and incremental value of MRS and NLR were evaluated using unadjusted Cox proportional hazards models in 85 patients (Supplementary Table S7). For DFS, MRS showed better discrimination than NLR alone (C-index, 0.750 vs. 0.683). The combined MRS + NLR model provided the highest discrimination and best model fit, with a C-index of 0.795 and an AIC of 446.9. Addition of NLR to the MRS model significantly improved model fit (LR χ^2^ = 12.342, df = 1, *p* < 0.001), whereas addition of MRS to the NLR model produced a larger improvement in discrimination and fit (ΔC-index = 0.113; LR χ^2^ = 50.278, df = 1, *p* < 0.001).

Similar findings were observed for OS. The combined MRS + NLR model had the highest C-index and lowest AIC among the evaluated models (C-index, 0.770; AIC, 446.4). Addition of NLR to MRS provided a significant incremental improvement (ΔC-index = 0.043; LR χ^2^ = 6.374, df = 1, *p* = 0.012), while addition of MRS to NLR resulted in a greater improvement (ΔC-index = 0.095; LR χ^2^ = 30.534, df = 1, *p* < 0.001). These findings indicate that MRS and NLR provided complementary prognostic information, although the incremental contribution of MRS beyond NLR was greater than that of NLR beyond MRS.

**Figure 3:**
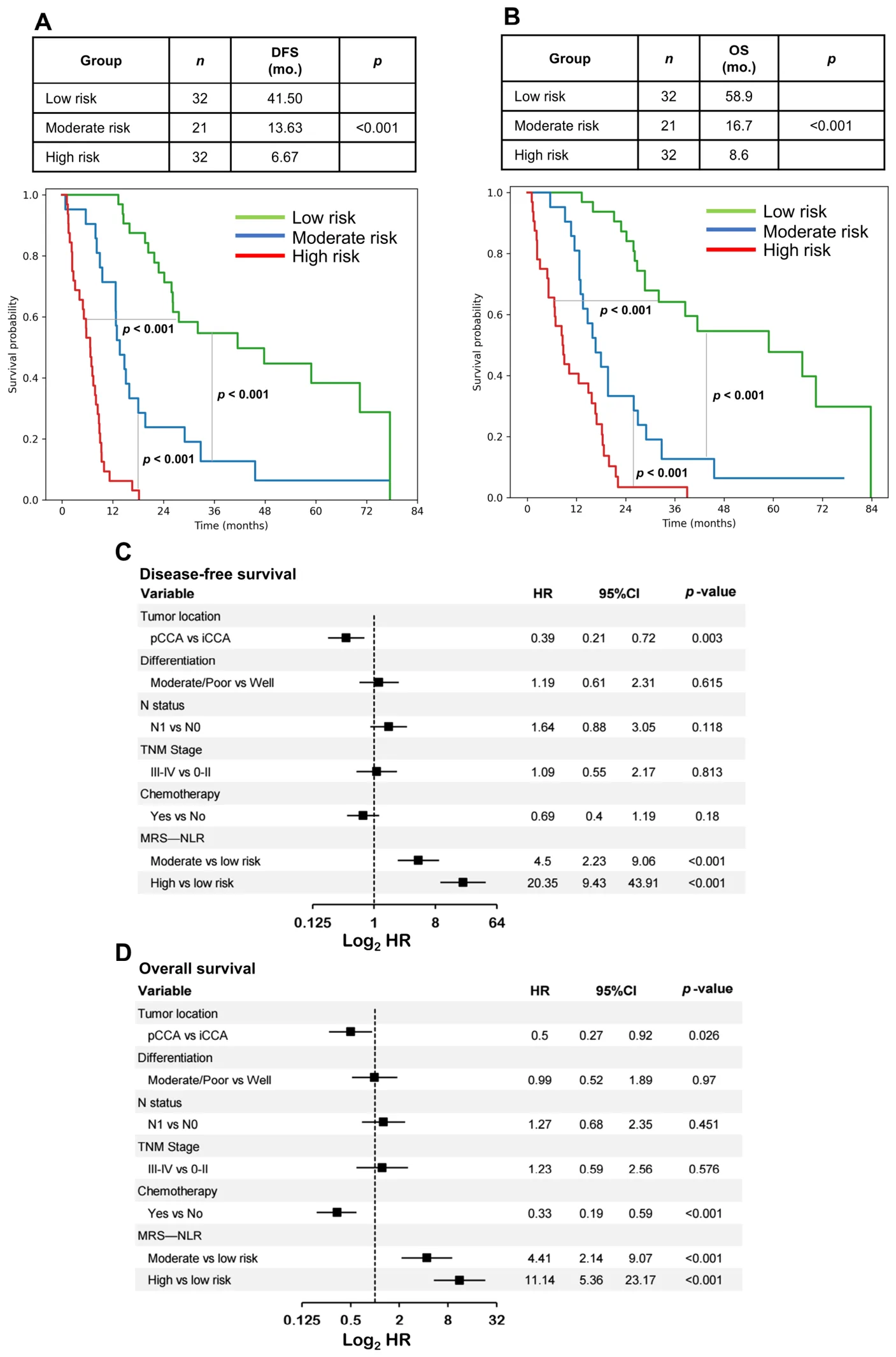
Combined MRS–NLR risk stratification for DFS and OS. Kaplan–Meier curves for DFS (**A**) and OS (**B**) according to combined MRS–NLR stratification. Multivariable Cox proportional hazards models for DFS (**C**) and OS (**D**) incorporated clinicopathological variables and combined MRS–NLR stratification. HRs and 95% CIs are shown, with the low-risk group used as the reference category. The vertical dashed line indicates HR = 1.0.

Based on these findings, patients were stratified into low-risk (low MRS/low NLR; *n* = 32, 38%), moderate-risk (discordant MRS and NLR; *n* = 21, 24%), and high-risk groups (high MRS/high NLR; *n* = 32, 38%). Kaplan–Meier analysis demonstrated apparent stepwise separation of DFS across the three groups (Figure 3A), with the high-risk group had the shortest DFS (log-rank *p* < 0.001). The moderate-risk group also had significantly poorer DFS than the low-risk group (*p* < 0.001). A similar pattern was observed for OS (Figure 3B), with significantly worse survival in the moderate- and high-risk groups compared with the low-risk group (*p* < 0.001).

Multivariable Cox analysis incorporating clinicopathological variables and combined MRS–NLR stratification is summarized in Figure 3C,D. For DFS, pCCA was associated with a lower recurrence hazard than iCCA (HR 0.39, 95% CI 0.21–0.72, *p* = 0.003). Compared with the low-risk group, the moderate-risk and high-risk MRS–NLR groups were associated with higher recurrence hazards, with adjusted HRs of 4.50 (95% CI 2.23–9.06, *p* < 0.001) and 20.35 (95% CI 9.43–43.91, *p* < 0.001), respectively. Other clinicopathological variables, including differentiation, nodal status, TNM stage, and adjuvant chemotherapy exposure, were not independently significant in the DFS model.

For OS, pCCA was also associated with a lower mortality hazard than iCCA (HR 0.50, 95% CI 0.27–0.92, *p* = 0.026), and adjuvant chemotherapy exposure was associated with a lower mortality hazard (HR 0.33, 95% CI 0.19–0.59, *p* < 0.001). Combined MRS–NLR stratification showed an adjusted association with mortality, with higher hazards observed in the moderate-risk group (HR 4.41, 95% CI 2.14–9.07, *p* < 0.001) and high-risk group (HR 11.14, 95% CI 5.36–23.17, *p* < 0.001) compared with the low-risk group. Differentiation, nodal status, and TNM stage were not independently significant after adjustment.

As additional diagnostic and sensitivity analyses for the combined MRS–NLR multivariable models, PH assumption testing using Schoenfeld residuals and ridge-penalized Cox regression sensitivity analysis were performed. For the DFS combined model, Schoenfeld residual testing showed no evidence of global PH violation (global χ^2^ = 4.482, df = 7, *p* = 0.723). Ridge-penalized Cox regression showed directionally consistent associations for all included variables, with the moderate- and high-risk MRS–NLR groups retaining the same direction of association after penalization (Supplementary Table S8). For the OS combined model, Schoenfeld residual testing suggested possible global PH deviation (global χ^2^ = 16.350, df = 7, *p* = 0.022), with possible deviations observed for the high-risk MRS–NLR group and adjuvant chemotherapy exposure. Therefore, HR estimates from the OS combined model were interpreted cautiously as average associations over follow-up. Ridge-penalized Cox regression showed directionally consistent associations for the MRS–NLR moderate- and high-risk groups after penalization (Supplementary Table S9).

In addition, exploratory subgroup analyses were performed to examine whether the outcome patterns associated with combined MRS–NLR stratification were observed across selected clinical strata, including tumor location and adjuvant chemotherapy exposure. Across iCCA and pCCA subgroups, higher MRS–NLR strata generally showed shorter DFS and OS, with broadly similar Kaplan–Meier patterns across risk groups (Supplementary Figure S1A–D). Similar patterns were also observed among patients who received and did not receive adjuvant chemotherapy (Supplementary Figure S1E–H).

Because these subgroup analyses involved smaller sample sizes and fewer events, subgroup-specific hazard ratio estimates were considered exploratory and should be interpreted cautiously. Full subgroup-specific Cox estimates, including HRs, 95% CIs, and *p*-values, are provided in Supplementary Tables S10–S13 for transparency. These analyses were intended to assess descriptive consistency of outcome patterns and should not be interpreted as definitive evidence of independent subgroup-specific prognostic effects or as evidence that MRS–NLR status predicts benefit from adjuvant chemotherapy.

Additional exploratory forest plot analyses using dichotomized MRS–NLR status showed broadly similar directions of association across selected clinicopathological strata, including tumor location, resection margin status, tumor morphology, differentiation, nodal status, TNM stage, and adjuvant chemotherapy exposure (Supplementary Figure S2). These findings should be regarded as supportive and hypothesis-generating because of limited subgroup sizes and wide confidence intervals in some strata.

## 4. Discussion

In this retrospective exploratory analysis, a parsimonious MRS-10 was associated with recurrence and survival outcomes in patients with resected CCA. The model was specifically developed to distinguish early from late recurrence among patients who experienced recurrence, rather than to predict the occurrence of recurrence in an unselected postoperative population. When evaluated together with the NLR, the combined MRS–NLR framework identified three prognostic groups with stepwise differences in DFS and OS. These findings suggest that postoperative prognosis in resected CCA may reflect complementary contributions from tumor-associated metabolic alterations and host systemic inflammatory status, in addition to conventional clinicopathological factors.

The MRS-10 model was derived from recurrence-associated metabolites identified in our previous serum metabolomics study [11]. Although the original metabolomic analysis identified a broader panel of approximately 90 metabolites associated with recurrence patterns, the present study focused on reducing this high-dimensional information into a smaller and more interpretable risk score. Among the candidate panel sizes evaluated, the 10-metabolite model provided a pragmatic balance between internally validated discrimination, model parsimony, specificity, and potential feasibility for targeted-assay development. However, its performance was comparable to that of the larger candidate panels, and the MRS-10 should not be interpreted as statistically superior or as the only valid metabolite combination. The selection of ten metabolites should therefore be interpreted as a strategy for model simplification rather than evidence that these metabolites alone fully represent the biology of recurrence. Instead, MRS-10 may serve as a surrogate summary of a broader recurrence-associated metabolic profile. This approach is consistent with the concept that cancer-related metabolic phenotypes often arise from coordinated pathway-level alterations rather than isolated metabolite changes [9,10]. Importantly, the internally validated performance estimates were substantially more conservative than the apparent development-cohort results. Repeated nested cross-validation was therefore considered the primary assessment of model discrimination, whereas the apparent performance was interpreted as optimistic. Feature-selection analysis also demonstrated incomplete stability of the full panel across resamples. Accordingly, the MRS-10 should be regarded as a parsimonious, data-derived candidate model rather than a definitive biological signature or an externally validated clinical prediction tool. Application in independent populations will require validation of the locked metabolite panel, preprocessing parameters, coefficients, calibration, and cutoff.

At the biological level, the metabolites retained in the MRS-10 model were related to pathways involved in amino acid metabolism, mitochondrial energy production, redox regulation, nucleotide turnover, and lipid remodeling. Alterations in branched-chain amino acid, glutamine, arginine, and tryptophan metabolism may reflect increased substrate utilization for biosynthesis, tricarboxylic acid cycle activity, and redox homeostasis [23]. Similar metabolic rewiring has been associated with aggressive tumor behavior and poor prognosis in hepatobiliary and gastrointestinal malignancies [24,25,26]. In particular, dysregulation of tryptophan metabolism and the kynurenine pathway has been linked to immune modulation, immune evasion, and tumor progression [27,28,29]. In parallel, alterations in mitochondrial metabolism, nucleotide turnover, and lipid-associated pathways may reflect metabolic flexibility and stress adaptation in tumors prone to recurrence [30,31,32,33]. These biological associations provide plausible context for the observed prognostic association of MRS-10, although mechanistic validation is required to clarify the functional roles of these metabolites in CCA progression.

A key observation in this study was the association between high MRS and elevated NLR. This finding suggests that adverse metabolic profiles may coexist with systemic inflammation in a subset of patients with more aggressive disease biology. The prognostic relevance of NLR in CCA has been reported in multiple studies, with elevated preoperative NLR associated with inferior DFS and OS [34,35,36]. Consistent with these reports, Chindaprasirt et al. showed that high NLR was associated with poorer survival outcomes in patients with resected intrahepatic CCA [21]. A meta-analysis including 4123 patients further supported elevated preoperative NLR as a marker of reduced overall and recurrence-free survival in CCA [22]. Biologically, elevated NLR may reflect neutrophil predominance and relative lymphocyte depletion. Neutrophils can support tumor progression through inflammatory cytokine release, angiogenic signaling, reactive oxygen species production, and extracellular matrix remodeling, whereas lymphopenia may indicate impaired antitumor immune surveillance [37]. Therefore, the combined MRS–NLR framework may capture both tumor-associated metabolic dysregulation and host inflammatory–immune status.

Experimental and translational studies provide additional support for a potential link between metabolic reprogramming and inflammation. Lipid-rich metabolic environments have been shown to suppress T-cell effector function and enhance neutrophil-associated reactive oxygen species production, potentially contributing to DNA damage, immune dysfunction, and tumor progression [12,38]. In CCA, interactions among metabolic dysregulation, inflammatory signaling, and immune evasion have also been implicated in disease progression [3,39]. Similar combined metabolic–inflammatory signatures have been reported in other malignancies, including gastric cancer, hepatocellular carcinoma, colorectal cancer, and endometrial cancer [14,40,41,42,43,44]. These observations support the broader concept that metabolic and inflammatory processes may interact as part of a tumor-promoting axis [10,45]. However, in the present study, these relationships should be interpreted as associative rather than causal.

The combined MRS–NLR framework provided additional prognostic stratification compared with MRS or NLR alone within the present dataset. The three-tier classification showed stepwise differences in DFS and OS, suggesting that integrating metabolic and inflammatory information may capture complementary biological dimensions of postoperative risk. From a potential clinical perspective, if externally validated, the combined framework could be used after curative-intent resection as an adjunct to conventional clinicopathological assessment. Patients classified into the high-risk group could potentially be considered for closer postoperative surveillance, including shorter follow-up intervals or earlier imaging, as well as multidisciplinary review and preferential enrollment in prospective clinical trials evaluating intensified or biomarker-guided adjuvant strategies. However, the present study does not demonstrate that altering surveillance intensity or adjuvant treatment according to MRS–NLR status improves patient outcomes. Therefore, the combined score should not currently be used to direct treatment selection or modify standard postoperative management. Prospective external validation, assessment of calibration and clinical utility, and evaluation of whether score-guided management improves outcomes are required before clinical implementation. However, the large hazard ratio estimates, particularly in the high-risk group, should be interpreted cautiously because they may partly reflect the modest sample size and event distribution. Additional model diagnostics were therefore performed to assess the proportional hazards assumption and the stability of multivariable Cox estimates. The main DFS and OS models evaluating MRS and NLR separately showed no evidence of global PH violation, and ridge-penalized Cox sensitivity analyses showed directionally consistent associations. For the combined MRS–NLR models, the DFS model satisfied the PH assumption, whereas the OS model showed possible global PH deviation. Accordingly, OS hazard ratio estimates from the combined model should be interpreted as average associations over follow-up rather than time-constant effects. Overall, the sensitivity analyses support the direction of the observed associations but should not be interpreted as external validation or confirmation of precise effect sizes.

Exploratory subgroup analyses showed broadly similar outcome patterns across tumor location and adjuvant chemotherapy exposure strata. CCA comprises anatomically and biologically distinct subtypes, with intrahepatic and perihilar tumors differing in cellular origin, molecular alterations, and clinical behavior [3,4]. Intrahepatic CCA is more frequently associated with IDH1/2 mutations and FGFR2 fusions, whereas perihilar CCA more commonly exhibits alterations involving pathways such as ERBB2, TP53, and KRAS, although reported frequencies vary across studies and etiologic backgrounds [5,6]. The observation that higher MRS–NLR strata were generally associated with shorter DFS and OS across tumor locations suggests that metabolic–inflammatory profiling may capture biological information that complements anatomical classification [46,47,48]. Nevertheless, these subgroup findings should be regarded as exploratory and hypothesis-generating because of limited subgroup sizes and wide confidence intervals. The relationship between MRS–NLR status and adjuvant chemotherapy exposure also requires cautious interpretation. In this exploratory analysis, patients with higher MRS–NLR status showed unfavorable outcomes in both chemotherapy-exposed and unexposed subgroups. However, this observation should not be interpreted as evidence of treatment resistance or reduced chemotherapy efficacy, as the present study was not designed or powered to evaluate predictive treatment effects. Adjuvant chemotherapy is an evidence- and guideline-supported treatment option in resected biliary tract cancer [49,50]. In retrospective cohorts, treatment exposure may be influenced by treatment-selection bias, postoperative recovery, performance status, comorbidities, pathological risk features, institutional practice, and physician discretion [51]. Therefore, these findings are more consistent with a prognostic association of MRS–NLR status rather than evidence of predictive value for adjuvant chemotherapy benefit. Although metabolic reprogramming has been linked to therapeutic resistance through enhanced antioxidant capacity, stress-response pathways, and metabolic flexibility [52,53], this remains a hypothesis-generating interpretation in the present study and requires prospective and functional validation in biliary tract cancer models.

Several limitations should be acknowledged. First, this was a single-center retrospective secondary analysis with a modest sample size and no independent external validation cohort. Although bootstrap validation and repeated nested cross-validation were performed, the MRS-10 should be regarded as an exploratory internally assessed model rather than a clinically validated tool. Second, the present cohort completely overlapped with the previously published metabolomic dataset from which the candidate metabolites were derived. Because the original candidate-selection process was informed by recurrence outcome in the same patients, residual selection-related optimism cannot be excluded, even though no previous coefficients, cutoffs, or data partitions were reused. Third, the high candidate predictor-to-sample-size ratio increased the risk of overfitting and feature-selection instability. The final MRS-10 should therefore be considered a parsimonious data-derived candidate panel rather than a unique or definitive biological signature. Fourth, dichotomization of continuous variables, including MRS, NLR, and age, may have reduced statistical information and obscured non-linear associations. The MRS cutoff was also derived within the present cohort and requires external validation or recalibration. Fifth, the multivariable and subgroup survival analyses were limited by sample size and event numbers. Some hazard-ratio estimates were large and imprecise, and possible non-proportional hazards in the combined OS model require cautious interpretation. Finally, adjuvant chemotherapy was treated as a fixed postoperative exposure, which may have introduced immortal-time and treatment-selection bias. Residual confounding from comorbidities, infection, perioperative factors, treatment selection, and recurrence management also cannot be excluded. No targeted assay, longitudinal profiling, or functional validation was performed. Prospective multicenter validation using a locked panel, coefficients, preprocessing procedure, and cutoff is required before clinical implementation.

## 5. Conclusions

In this retrospective exploratory reanalysis, MRS-10 was associated with recurrence timing and survival outcomes among patients with resected CCA who experienced recurrence. When combined with preoperative NLR, the MRS–NLR framework stratified patients into groups with stepwise differences in DFS and OS, suggesting that metabolic and systemic inflammatory information may provide complementary prognostic context alongside conventional clinicopathological factors. If independently validated, this framework could potentially support postoperative risk stratification and help identify patients who may warrant closer surveillance, multidisciplinary assessment, or prioritization for prospective clinical trials. However, the present findings do not support using MRS–NLR status to guide adjuvant treatment or alter standard clinical management. Given the retrospective design, modest sample size, cohort overlap with the previous metabolomic analysis, and absence of independent external validation, these findings should be regarded as hypothesis-generating. Larger independent and prospective studies are required before MRS-10 or the combined MRS–NLR framework can be considered for clinical application.

## Figures and Tables

**Table 1:** Nested cross-validation performance of candidate metabolite panels considered for final model selection.

Model	Pooled OOF AUC (95% CI)	Mean Outer-Fold AUC ± SD	Youden Cutoff	SEN.	SPC.	ACC.
MRS-17	0.833 (0.730–0.903)	0.796 ± 0.089	0.196	0.946	0.667	0.784
MRS-12	0.830 (0.727–0.904)	0.817 ± 0.083	0.116	0.919	0.686	0.784
MRS-10	0.828 (0.725–0.911)	0.819 ± 0.089	0.465	0.757	0.804	0.784
MRS-8	0.803 (0.702–0.894)	0.795 ± 0.080	0.088	0.918	0.686	0.784

Table footnote: Values are presented as the pooled patient-level out-of-fold (OOF) area under the receiver operating characteristic curve (AUC) with 95% confidence intervals or as the mean outer-fold AUC ± standard deviation. Pooled patient-level OOF AUCs were calculated using the aggregated OOF predicted probabilities, whereby each patient was evaluated using models that did not include that patient in the corresponding training set. The optimal probability cutoff for each model was determined by maximizing the Youden index on the pooled OOF predictions. Sensitivity, specificity, and accuracy were calculated at the corresponding Youden cutoff. Early recurrence was defined as the positive class and late recurrence as the negative class. Abbreviations: ACC, accuracy; AUC, area under the receiver operating characteristic curve; MRS, metabolite risk score; OOF, out-of-fold; SEN, sensitivity; SPC, specificity.

**Table 2:** Association between clinicopathological features and metabolite-based risk score.

Variable	Low MRS, *n* (%)	High MRS, *n* (%)	*p*-Value
Age, years (median cutoff)			
<62	21 (50)	18 (41.9)	0.451
≥62	21 (50)	25 (58.1)	
Gender			
Male	15 (35.7)	16 (37.2)	0.886
Female	27 (64.3)	27 (62.8)	
Tumor location			
iCCA	24 (57.1)	26 (60.5)	0.928
pCCA	18 (42.9)	17 (39.5)	
R status			
R0	29 (69.0)	20 (46.5)	0.060
R1	13 (31.0)	23 (53.5)	
Tumor morphology			
ID	8 (19.0)	1 (2.3)	0.041
ID mix	10 (23.8)	14 (32.6)	
Without ID	24 (57.1)	28 (65.1)	
Differentiation			
Well	36 (85.7)	31 (72.1)	0.204
Moderate/poor	6 (14.3)	12 (27.9)	
N stage			
N0	29 (69.0)	19 (44.2)	0.036
N1	13 (31.0)	24 (55.8)	
Stage			
0–II	13 (31.0)	6 (14.0)	0.105
III–IV	29 (69.0)	37 (86.0)	
NLR			
Low	32 (76.2)	11 (25.6)	<0.001
High	10 (23.8)	32 (74.4)	
Adjuvant chemotherapy			
Yes	32 (76.2)	22 (51.2)	0.030
No	10 (23.8)	21 (48.8)	

**Table 3:** Univariate and multivariate analyses of disease-free survival and overall survival according to clinicopathological factors and metabolic risk score.

Variable	*n*	DFS (Month)	Univariate	Multivariate		OS (Month)	Univariate	Multivariate	
			*p*-Value	HR (95%CI)	*p*-Value		*p*-Value	HR (95%CI)	*p*-Value
Age, years (median cutoff)									
<62	39	14.7		-		21.6		-	
≥62	46	13.3	0.230	-	-	18.4	0.228	-	-
Gender									
Male	31	14.5		-		22.9		-	
Female	54	13.5	0.805	-	-	18.7	0.448	-	-
Tumor location									
iCCA	50	12.7		1		18.7		1	
pCCA	35	15.9	0.142	0.42 (0.23–0.78)	0.006	25.9	0.120	0.57 (0.32–1.01)	0.053
R status									
R0	49	18		-		25.9		-	
R1	36	9.9	0.556	-	-	15.9	0.308	-	-
Tumor morphology									
ID mix	33	14.5		-		27		1	
Without ID	52	12.9	0.402	-	-	18.2	0.191	1.21 (0.66–2.22)	0.528
Differentiation									
Well	67	14.5		1		22.1		1	
Moderate/poor	18	12.7	0.181	0.96 (0.47–1.95)	0.911	16.7	0.167	0.87 (0.43–1.76)	0.694
N stage									
N0	48	19.7		1		25.9		1	
N1	37	8.8	0.012	1.62 (0.85–3.09)	0.144	15.9	0.016	1.15 (0.61–2.17)	0.662
Stage									
0–II	19	26.2		1		38.5		1	
III–IV	66	12.7	0.045	1.07 (0.54–2.11)	0.854	16.9	0.026	1.23 (0.60–2.55)	0.574
NLR									
Low	43	26.3		1		32.1			
High	42	7.8	<0.001	2.84 (1.55–5.20)	0.001	12.7	<0.001	2.75 (1.46–5.15)	0.002
Adjuvant chemotherapy									
No	31	9		1		10.2			
Yes	54	18	0.019	0.79 (0.45–1.39)	0.406	25.9	<0.001	0.32 (0.18–0.59)	<0.001
MRS									
Low MRS	42	32		1		38.5		1	
High MRS	43	7.4	<0.001	7.92 (3.94–15.92)	<0.001	10.7	<0.001	4.05 (2.08–7.89)	<0.001

Abbreviations: DFS, disease-free survival; OS, overall survival; HR, hazard ratio; CI, confidence interval; iCCA, intrahepatic cholangiocarcinoma; pCCA, perihilar cholangiocarcinoma; NLR, neutrophil-to-lymphocyte ratio; MRS, metabolite-based risk score. For univariate analyses, median survival times and *p*-values are shown. Variables with *p* < 0.20 in univariate analysis were entered into the multivariate Cox proportional hazards models. Multivariate HRs and 95% CIs are shown for variables included in the adjusted models. Dash symbols indicate variables that were not included in the multivariate model because they did not meet the predefined selection criterion.

## Data Availability

The data analyzed in this study are available on request from the corresponding author due to ethical and privacy restrictions related to patient-level clinical data. The data were derived from a previously published cohort [11] (https://doi.org/10.1038/s41598-025-97641-9).

## Supplementary Materials

The following supporting information can be downloaded at: https://www.mdpi.com/article/10.3390/medsci14050525/s1, Figure S1: Exploratory Kaplan–Meier analyses of combined MRS–NLR stratification according to tumor location and adjuvant chemotherapy exposure. Kaplan–Meier curves for DFS and OS are shown in iCCA (A–B), pCCA (C–D), patients receiving adjuvant chemotherapy (E–F), and patients not receiving adjuvant chemotherapy (G–H). Patients were stratified into low-, moderate-, and high-risk groups according to combined MRS–NLR status. Survival differences were assessed using the log-rank test, with *p*-values shown in each panel. These subgroup analyses were exploratory and should be interpreted cautiously because of limited sample sizes and event numbers in some strata; Figure S2: Exploratory subgroup forest plots for DFS and OS according to clinicopathological variables. Hazard ratios and 95% confidence intervals are shown for moderate/high MRS–NLR status compared with low-risk status within selected clinicopathological subgroups. The vertical dashed line indicates HR = 1.0. These analyses were exploratory and were intended to assess the direction of associations across clinical strata. Subgroup-specific estimates should be interpreted cautiously because of limited sample sizes, limited event numbers, and wide confidence intervals in some strata. Table S1: LASSO model development and regularization parameter selection; Table S2: Bootstrap internal validation of the MRS-10 model; Table S3: Repeated nested cross-validation performance of the MRS-10 model; Table S4: Final MRS-10 metabolites, coefficients, and score equation; Table S5: Proportional hazards assumption and ridge-penalized Cox sensitivity analysis for the DFS multivariable model; Table S6: Proportional hazards assumption and ridge-penalized Cox sensitivity analysis for the OS multivariable model; Table S7: Prognostic performance and incremental value of MRS and NLR for DFS and OS; Table S8: Proportional hazards assumption and ridge-penalized Cox sensitivity analysis for the DFS combined MRS–NLR multivariable model; Table S9: Proportional hazards assumption and ridge-penalized Cox sensitivity analysis for the OS combined MRS–NLR multivariable model; Table S10: Subgroup analysis of DFS and OS according to combined MRS and NLR in patients with iCCA; Table S11: Subgroup analysis of DFS and OS according to combined MRS and NLR in patients with pCCA; Table S12: Subgroup analysis of DFS and OS according to combined MRS and NLR in CCA patients receiving chemotherapy; Table S13: Subgroup analysis of DFS and OS according to combined MRS and NLR in CCA patients not receiving chemotherapy.

## Abbreviations

The following abbreviations are used in this manuscript:

AIC	Akaike Information Criterion
AJCC	American Joint Committee on Cancer
AUC	Area under the curve
CCA	Cholangiocarcinoma
CI	Confidence interval
DFS	Disease-free survival
FDR	False Discovery Rate
HR	Hazard ratio
iCCA	Intrahepatic cholangiocarcinoma
LASSO	Least absolute shrinkage and selection operator
LC–MS/MS	Liquid chromatography–tandem mass spectrometry
LR	Likelihood Ratio
MRS	Metabolite-based risk score
NLR	Neutrophil-to-lymphocyte ratio
OOF	Out-of-Fold
OS	Overall survival
pCCA	Perihilar cholangiocarcinoma
PH	Proportional Hazards
ROC–AUC	Area under the receiver operating characteristic curve
TNM	Tumor–node–metastasis
