# Supplementary material for: Metabolic and Inflammatory Risk Stratification in Resected Cholangiocarcinoma: A Retrospective Exploratory Analysis
Source: Med Sci (Basel). 2026 Aug 28;14(5):525. doi: 10.3390/medsci14050525 (PMC13609717; doi:10.3390/medsci14050525)
Supplement: Supplementary file 1 [file medsci-14-00525-s001.zip › medsci-4444250-supplementary.pdf]

Supplementary Materials: Metabolic and Inflammatory Risk Stratification in Resected Cholangiocarcinoma: A Retrospective Exploratory Analysis

Piya Prajumwongs, Attapol Titapun, Vasin Thanasukarn, Apiwat Jareanrat, Natcha Khuntikeo, Nisana Namwat, Poramate Klanrit, Arporn Wangwiwatsin, Jarin Chindaprasirt, Prakasit Sa-Ngiamwibool, Nattha Muangritdech, Sittiruk Roytrakul and Watcharin Loilome

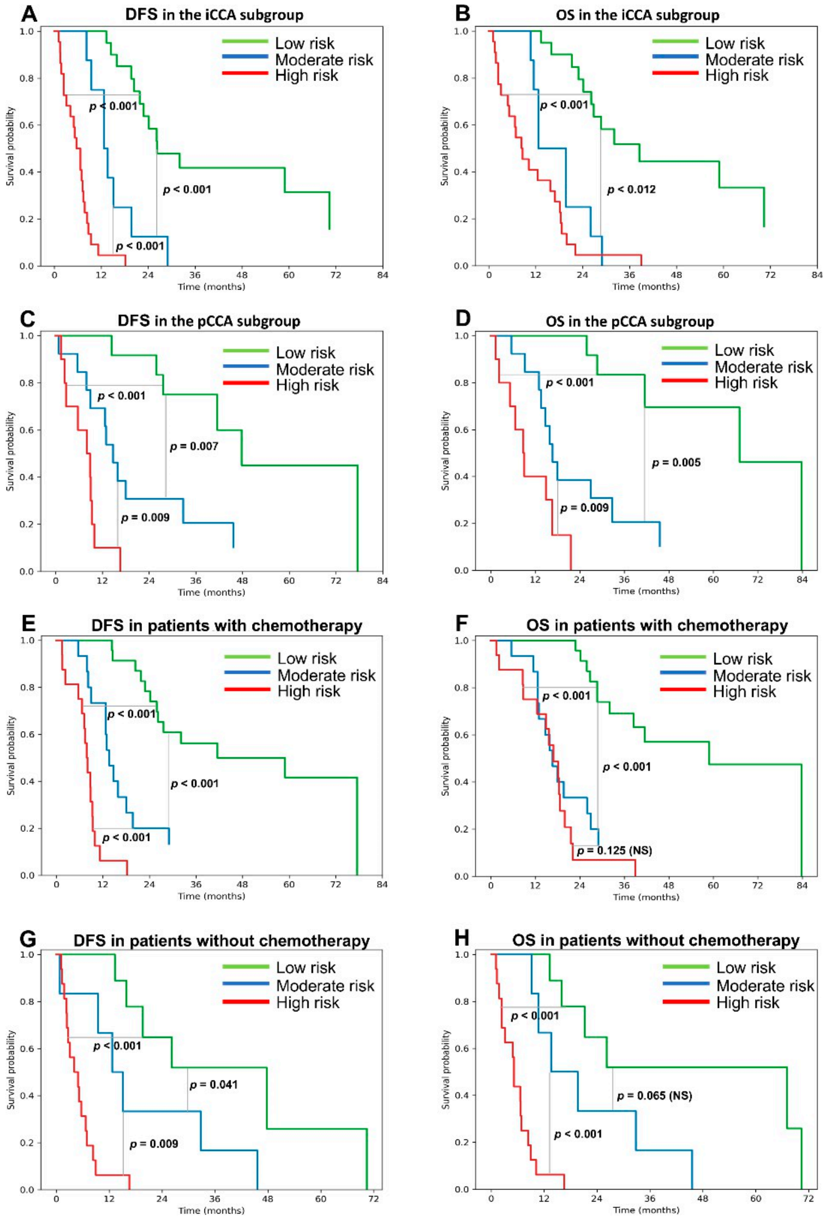

Supplementary Figure S1. Exploratory Kaplan–Meier analyses of combined MRS–NLR stratification according to tumor location and adjuvant chemotherapy exposure. Kaplan–Meier curves for DFS and OS

are shown in iCCA (A–B), pCCA (C–D), patients receiving adjuvant chemotherapy (E–F), and patients not receiving adjuvant chemotherapy (G–H). Patients were stratified into low-, moderate-, and high-risk groups according to combined MRS–NLR status. Survival differences were assessed using the log-rank test, with p-values shown in each panel. These subgroup analyses were exploratory and should be interpreted cautiously because of limited sample sizes and event numbers in some strata.

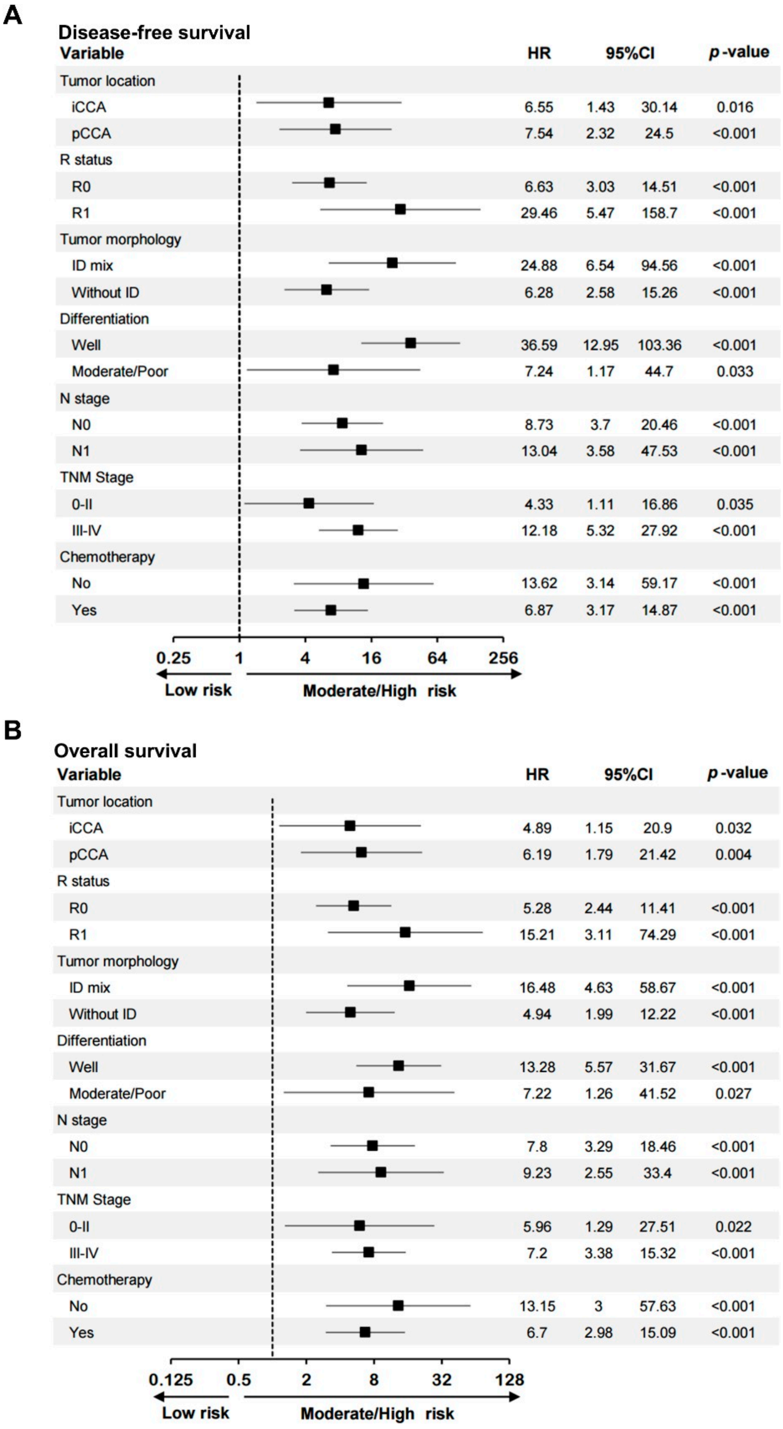

A
Disease-free survival
Variable
HR
95%CI
p -value
Tumor location
iCCA 6.55 1.43 30.14 0.016
pCCA 7.54 2.32 24.5 <0.001
R status
R0 6.63 3.03 14.51 <0.001
R1 29.46 5.47 158.7 <0.001
Tumor morphology
ID mix 24.88 6.54 94.56 <0.001
Without ID 6.28 2.58 15.26 <0.001
Differentiation
Well 36.59 12.95 103.36 <0.001
Moderate/Poor 7.24 1.17 44.7 0.033
N stage
N0 8.73 3.7 20.46 <0.001
N1 13.04 3.58 47.53 <0.001
TNM Stage
0-II 4.33 1.11 16.86 0.035
III-IV 12.18 5.32 27.92 <0.001
Chemotherapy
No 13.62 3.14 59.17 <0.001
Yes 6.87 3.17 14.87 <0.001
0.25 1 4 16 64 256
Low risk
Moderate/High risk
B
Overall survival
Variable
HR
95%CI
p -value
Tumor location
iCCA 4.89 1.15 20.9 0.032
pCCA 6.19 1.79 21.42 0.004
R status
R0 5.28 2.44 11.41 <0.001
R1 15.21 3.11 74.29 <0.001
Tumor morphology
ID mix 16.48 4.63 58.67 <0.001
Without ID 4.94 1.99 12.22 <0.001
Differentiation
Well 13.28 5.57 31.67 <0.001
Moderate/Poor 7.22 1.26 41.52 0.027
N stage
N0 7.8 3.29 18.46 <0.001
N1 9.23 2.55 33.4 <0.001
TNM Stage
0-II 5.96 1.29 27.51 0.022
III-IV 7.2 3.38 15.32 <0.001
Chemotherapy
No 13.15 3 57.63 <0.001
Yes 6.7 2.98 15.09 <0.001
0.125 0.5 2 8 32 128
Low risk
Moderate/High risk

Supplementary Figure S2. Exploratory subgroup forest plots for DFS and OS according to clinicopathological variables. Hazard ratios and 95% confidence intervals are shown for moderate/high MRS–NLR status compared with low-risk status within selected clinicopathological subgroups. The vertical dashed line indicates HR = 1.0. These analyses were exploratory and were intended to assess the direction of associations across clinical strata. Subgroup-specific estimates should be interpreted cautiously because of limited sample sizes, limited event numbers, and wide confidence intervals in some strata.

Supplementary Table S1. LASSO model development and regularization parameter selection.

Step	Description	Result
Initial candidate metabolites	Metabolites entered into LASSO logistic regression	90
Preprocessing	Metabolite intensities were transformed using log10(x + 1) and standardized using Z-score normalization	Applied
Model	L1-penalized logistic regression	LASSO
Inner cross-validation	Stratified 5-fold cross-validation used for regularization-parameter evaluation	Applied
Regularization grid	Candidate C values were evaluated across a predefined grid, where C represents the inverse of the regularization strength	Applied
Best cross-validated model	Model with the highest mean inner-CV AUC	Best C = 0.0858; mean AUC = 0.8995; SD = 0.0614; SE = 0.0274
Regularization-parameter selection criterion	C values within one SE of the maximum mean inner-CV AUC were retained; among these, the C yielding a mean number of non-zero metabolites closest to approximately 17 was selected, with ties favoring the smaller C	Applied
Selected regularization parameter	Parameter used for final LASSO feature selection	C = 0.3981; λ = 2.5119; log10(λ) = 0.400
Mean CV AUC at selected C	Mean inner-CV AUC at the selected regularization parameter	0.8755
Variability at selected C	Variability in inner-CV AUC at the selected regularization parameter	SD = 0.0270; SE = 0.0121
Mean non-zero metabolites at selected C	Average number of retained metabolites across inner-CV folds	15.6
Non-zero metabolites after final LASSO fitting	Features retained after refitting LASSO on the full analytical dataset using the selected regularization parameter	17
Final simplified panel	Metabolites retained for the final parsimonious MRS-10 panel	10

Table footnote: Metabolite intensities were transformed using log10(x + 1) and standardized using Z-score normalization before LASSO logistic regression. Candidate values of *C*, the inverse of the regularization strength, were evaluated using stratified five-fold inner cross-validation. The value of *C* associated with the

highest mean inner-CV AUC was 0.0858. The final regularization parameter was selected using a two-stage performance-constrained rule: values of C within one standard error of the maximum mean inner-CV AUC were retained, and the value yielding a mean number of non-zero metabolites closest to the prespecified target of approximately 17 was selected, with ties favoring the smaller C. This procedure selected $C = 0.3981$, corresponding to $\lambda = 1/C = 2.5119$. Refitting the LASSO model on the full analytical dataset retained 17 non-zero metabolites, from which the final parsimonious MRS-10 panel was derived.

Abbreviations: AUC, area under the receiver operating characteristic curve; CV, cross-validation; LASSO, least absolute shrinkage and selection operator; MRS, metabolite risk score; SD, standard deviation; SE, standard error.

Supplementary Table S2. Bootstrap internal validation of the MRS-10 model.

Metric	Value
Apparent AUC	0.973
Mean optimism in AUC	0.07
Optimism-corrected AUC	0.903
Bootstrap test-on-original AUC, mean ± SD	0.928 ± 0.023
Bootstrap test-on-original AUC, 95% interval	0.880–0.962
Apparent Brier score	0.059
Mean optimism in Brier score	0.089
Optimism-corrected Brier score	0.148
Apparent calibration intercept	−0.0002
Mean optimism in calibration intercept	0.149
Optimism-corrected calibration intercept	−0.149
Apparent calibration slope	1.001

Abbreviations: AUC, area under the receiver operating characteristic curve; MRS, metabolite-based risk score; SD, standard deviation.

Notes: Bootstrap internal validation was performed using 200 bootstrap resamples. In each bootstrap iteration, the complete MRS-10 modelling pipeline, including preprocessing, LASSO-based feature selection, metabolite ranking, final model fitting, and performance evaluation, was repeated. Optimism was calculated as the difference between model performance in the bootstrap sample and performance when the bootstrap-derived model was tested on the original dataset. The optimism-corrected AUC was calculated as the apparent AUC minus the mean estimated optimism. Calibration intercept and calibration slope were estimated using a logistic calibration model based on the logit of the predicted probability. A calibration slope lower than 1 indicates potential overfitting or overconfident predicted probabilities.

Supplementary Table S3. Repeated nested cross-validation performance of the MRS-10 model.

Metric	Value
Pooled patient-level OOF AUC (95%CI)	0.828 (0.725–0.911)
Mean outer-fold AUC ± SD	0.819 ± 0.089
Median outer-fold AUC	0.844
Outer-fold AUC IQR	0.750–0.888
Mean outer-fold Brier score	0.237
SD of outer-fold Brier score	0.101
Number of valid outer folds	25

Notes: Repeated nested cross-validation was performed using stratified five-fold outer cross-validation with five repeats, resulting in 25 outer test folds. Within each outer-loop iteration, all data-dependent steps, including preprocessing, LASSO regularization-parameter selection, feature selection, metabolite ranking, and model fitting, were performed using only the corresponding outer training data. The held-out outer test fold was used exclusively for performance evaluation. Values in this table summarize performance across the 25 outer test folds. The Brier score measures the mean squared difference between predicted probabilities and observed outcomes, with lower values indicating better probabilistic prediction performance.

Abbreviations: AUC, area under the receiver operating characteristic curve; CV, cross-validation; IQR, interquartile range; LASSO, least absolute shrinkage and selection operator; MRS, metabolite risk score; SD, standard deviation.

Supplementary Table S4. Final MRS-10 metabolites, coefficients, and score equation.

Rank	Metabolite	Final Logistic Coefficient	Absolute Coefficient	Direction	LASSO Coefficient for Ranking
1	L-Threonine	-2.03197	2.03197	Negative	-0.52449
2	LysoPC(18:3(9Z,12Z,15Z)/0:0)	-1.77333	1.77333	Negative	-1.08863
3	PC(P-18:0/22:4(7Z,10Z,13Z,16Z))	-1.68616	1.68616	Negative	-0.30601
4	Palmitic acid	-1.30568	1.30568	Negative	-0.22553
5	LysoPC(16:1/0:0)	-1.30446	1.30446	Negative	-0.55274
6	LysoPC(14:0/0:0)	-1.09068	1.09068	Negative	-0.25019
7	SM(d18:0/24:1(15Z))	-0.93429	0.93429	Negative	-0.1914
8	Thymidine 5′-triphosphate	-0.70473	0.70473	Negative	-0.1789
9	Indoleacetaldehyde	0.46932	0.46932	Positive	0.1739
10	Linoleyl carnitine	0.29631	0.29631	Positive	0.2871

Abbreviations: MRS, metabolite-based risk score; LysoPC, lysophosphatidylcholine; PC, phosphatidylcholine; SM, sphingomyelin.

Notes: Metabolite intensities were transformed using log10(x+1) and standardized by Z-score normalization before calculation of the MRS-10. The MRS-10 linear predictor was calculated as $\beta_0 + \Sigma\beta_i Z_i$, where β_0 is the model intercept, β_i is the final logistic regression coefficient for each metabolite, and Z_i is the standardized log-transformed metabolite intensity. The model intercept was −1.62081. Absolute coefficients indicate the relative contribution of each metabolite to the final model.

MRS-10 linear predictor = −1.62081 − 2.03197 × Z(L-Threonine) − 1.77333 × Z[LysoPC(18:3(9Z,12Z,15Z)/0:0)] − 1.68616 × Z[PC(P-18:0/22:4(7Z,10Z,13Z,16Z))] − 1.30568 × Z(Palmitic acid) − 1.30446 × Z[LysoPC(16:1/0:0)] − 1.09068 ×

Z[LysoPC(14:0/0:0)] − 0.93429 × Z[SM(d18:0/24:1(15Z))] − 0.70473 × Z(Thymidine 5′-triphosphate) + 0.46932 × Z(Indoleacetaldehyde) + 0.29631 × Z(Linoleyl carnitine).

Predicted probability = 1/[1 + exp(−MRS-10 linear predictor)].

Z indicates the standardized log-transformed metabolite intensity after log10(x+1) transformation and Z-score normalization.

Supplementary Table S5. Proportional hazards assumption and ridge-penalized Cox sensitivity analysis for the DFS multivariable model.

Variable	Standard Cox HR	95% CI	Schoenfeld χ^2	PH p-value	PH interpretation	Ridge HR	Direction consistent	HR attenuated after penalization
MRS	7.92	3.94–15.92	1.803	0.179	PH assumption satisfied	7.56	Yes	Yes
Chemo	0.79	0.45–1.39	0.353	0.552	PH assumption satisfied	0.79	Yes	No
N	1.62	0.85–3.09	0.121	0.728	PH assumption satisfied	1.59	Yes	Yes
Location: pCCA vs iCCA	0.42	0.23–0.78	0.516	0.472	PH assumption satisfied	0.43	Yes	Yes
Stage	1.07	0.54–2.11	0.416	0.519	PH assumption satisfied	1.08	Yes	No
NLR	2.84	1.55–5.20	0.013	0.908	PH assumption satisfied	2.81	Yes	Yes
Differentiation	0.96	0.47–1.95	0.008	0.93	PH assumption satisfied	0.97	Yes	Yes

Global Schoenfeld PH test: $\chi^2 = 3.231$, df = 7, p = 0.863; PH assumption satisfied.

Abbreviations: CI, confidence interval; DFS, disease-free survival; HR, hazard ratio; MRS, metabolite-based risk score; NLR, neutrophil-to-lymphocyte ratio; pCCA, perihilar cholangiocarcinoma; PH, proportional hazards.

Note: Ridge-penalized Cox regression was performed as a sensitivity analysis. Direction consistent indicates that the direction of association was the same in standard Cox and ridge-penalized Cox models.

Supplementary Table S6. Proportional hazards assumption and ridge-penalized Cox sensitivity analysis for the OS multivariable model.

Variable	Standard Cox HR	95% CI	Schoenfeld χ^2	PH p-value	PH interpretation	Ridge HR	Direction consistent	HR attenuated after penalization
MRS	4.05	2.08–7.89	3.186	0.074	PH assumption satisfied	3.96	Yes	Yes
Chemo	0.32	0.18–0.59	2.476	0.116	PH assumption satisfied	0.33	Yes	Yes
N	1.15	0.61–2.17	1.056	0.304	PH assumption satisfied	1.15	Yes	Yes
Growth	1.21	0.66–2.22	0.92	0.337	PH assumption satisfied	1.2	Yes	Yes
Location: pCCA vs iCCA	0.57	0.32–1.01	1.883	0.17	PH assumption satisfied	0.57	Yes	Yes
Stage	1.23	0.60–2.55	0.12	0.729	PH assumption satisfied	1.23	Yes	No
NLR	2.75	1.46–5.15	0.459	0.498	PH assumption satisfied	2.7	Yes	Yes
Differentiation	0.87	0.43–1.76	0.518	0.472	PH assumption satisfied	0.88	Yes	Yes

Global Schoenfeld PH test: $\chi^2 = 10.617$, df = 8, p = 0.224; PH assumption satisfied.

Abbreviations: CI, confidence interval; HR, hazard ratio; MRS, metabolite-based risk score; NLR, neutrophil-to-lymphocyte ratio; OS, overall survival; pCCA, perihilar cholangiocarcinoma; PH, proportional hazards.

Note: Ridge-penalized Cox regression was performed as a sensitivity analysis. Direction consistent indicates that the direction of association was the same in the standard Cox and ridge-penalized Cox models.

Supplementary Table S7. Prognostic performance and incremental value of MRS and NLR for DFS and OS.

Outcome	Model comparison	C-index	AIC	ΔC-index†	ΔAIC‡	LR χ^2	df	p-value§
DFS	MRS	0.75	457.3	—	—	—	—	<0.001¶
DFS	NLR	0.683	495.2	—	—	—	—	<0.001¶
DFS	MRS + NLR	0.795	446.9	—	—	—	—	<0.001¶
DFS	MRS vs MRS + NLR	—	—	0.045	10.342	12.342	1	<0.001
DFS	NLR vs MRS + NLR	—	—	0.113	48.278	50.278	1	<0.001
OS	MRS	0.727	450.7	—	—	—	—	<0.001¶
OS	NLR	0.675	474.9	—	—	—	—	<0.001¶
OS	MRS + NLR	0.77	446.4	—	—	—	—	<0.001¶
OS	MRS vs MRS + NLR	—	—	0.043	4.374	6.374	1	0.012
OS	NLR vs MRS + NLR	—	—	0.095	28.534	30.534	1	<0.001

Abbreviations: AIC, Akaike information criterion; DFS, disease-free survival; LR, likelihood ratio; MRS, metabolite-based risk score; NLR, neutrophil-to-lymphocyte ratio; OS, overall survival.

Notes: All models were fitted in the same 85 patients, including 70 DFS events and 67 OS events. Higher C-index indicates better discrimination, whereas lower AIC indicates better model fit after accounting for model complexity.

† ΔC-index was calculated as the C-index of the combined MRS + NLR model minus that of the reduced model.

‡ ΔAIC was calculated as the AIC of the reduced model minus that of the combined model; positive values favor the combined model.

§ Nested-model p-values were obtained using likelihood-ratio tests comparing the combined model directly with the corresponding reduced model.

¶ For the individual Cox models, p-values were derived from the global likelihood-ratio test.

Supplementary Table S8. Proportional hazards assumption and ridge-penalized Cox sensitivity analysis for the DFS combined MRS–NLR multivariable model.

Variable	Standard Cox HR	95% CI	Schoenfeld χ^2	PH p-value	PH interpretation	Ridge HR	Direction consistent	HR attenuated after penalization
MRS–NLR moderate vs low	4.5	2.23–9.06	1.582	0.208	PH assumption satisfied	4.15	Yes	Yes
MRS–NLR high vs low	20.35	9.43–43.91	1.668	0.196	PH assumption satisfied	18.25	Yes	Yes
Chemotherapy: yes vs no	0.69	0.40–1.19	0.41	0.522	PH assumption satisfied	0.69	Yes	Yes
N stage: N1 vs N0	1.64	0.88–3.05	0.074	0.786	PH assumption satisfied	1.61	Yes	Yes
Location: pCCA vs iCCA	0.39	0.21–0.72	0.528	0.467	PH assumption satisfied	0.4	Yes	Yes
Stage: late vs early	1.09	0.55–2.17	0.199	0.655	PH assumption satisfied	1.1	Yes	No
Differentiation	1.19	0.61–2.31	0.02	0.886	PH assumption satisfied	1.19	Yes	No

Global Schoenfeld PH test: $\chi^2 = 4.482$, df = 7, p = 0.723; PH assumption satisfied.

Abbreviations: CI, confidence interval; DFS, disease-free survival; HR, hazard ratio; MRS, metabolite-based risk score; NLR, neutrophil-to-lymphocyte ratio; pCCA, perihilar cholangiocarcinoma; PH, proportional hazards.
Note: Ridge-penalized Cox regression was performed as a sensitivity analysis. Direction consistent indicates that the direction of association was the same in the standard Cox and ridge-penalized Cox models.

Supplementary Table S9. Proportional hazards assumption and ridge-penalized Cox sensitivity analysis for the OS combined MRS–NLR multivariable model.

Variable	Standard Cox HR	95% CI	Schoenfeld χ^2	PH p-value	PH interpretation	Ridge HR	Direction consistent	HR attenuated after penalization
MRS–NLR moderate vs low	4.41	2.14–9.07	1.207	0.272	PH assumption satisfied	4.11	Yes	Yes
MRS–NLR high vs low	11.14	5.36–23.17	6.104	0.013	Possible PH deviation	10.24	Yes	Yes
Chemotherapy: yes vs no	0.33	0.19–0.59	4.616	0.032	Possible PH deviation	0.35	Yes	Yes
N stage: N1 vs N0	1.27	0.68–2.35	1.103	0.294	PH assumption satisfied	1.26	Yes	Yes
Location: pCCA vs iCCA	0.5	0.27–0.92	2.025	0.155	PH assumption satisfied	0.51	Yes	Yes
Stage: late vs early	1.23	0.59–2.56	0.017	0.897	PH assumption satisfied	1.24	Yes	No
Differentiation	0.99	0.52–1.89	1.277	0.258	PH assumption satisfied	1	No	Yes

Global Schoenfeld PH test: $\chi^2 = 16.350$, df = 7, p = 0.022; possible global PH deviation.

Abbreviations: CI, confidence interval; HR, hazard ratio; MRS, metabolite-based risk score; NLR, neutrophil-to-lymphocyte ratio; OS, overall survival; pCCA, perihilar cholangiocarcinoma; PH, proportional hazards.

Note: Ridge-penalized Cox regression was performed as a sensitivity analysis. Direction consistent indicates that the direction of association was the same in the standard Cox and ridge-penalized Cox models.

Supplementary Table S10. Subgroup analysis of DFS and OS according to combined MRS and NLR in patients with iCCA.

**Variable**	*n*	DFS (month)	Univariate	Multivariate		OS (month)	Univariate	Multivariate	
			p-value	HR (95%CI)	*p*-value		*p*-value	HR (95%CI)	*p*-value
**R status**									
R0	32	13.3		-		19.9		-	
R1	18	9.4	0.583	-	-	12.7	0.350	-	-
**Tumor morphology**									
ID mix	18	13.6		-		26		-	-
Without ID	32	12.7	0.609	-	-	18.4	0.565	-	-
**Differentiation**									
Well	41	14.5		1		19.9		1	
Moderate/poor	9	4.9	0.003	4.07 (1.67–9.93)	0.002	12.7	0.035	1.65 (0.70–3.89)	0.249
**N stage**									
N0	34	15.1		1		19.9		1	
N1	16	7.4	0.007	1.59 (0.71–3.59)	0.261	15.7	0.008	1.17 (0.50–2.74)	0.711
**Stage**									
0-II	11	22.9		1		29.0		1	
III-IV	39	11.3	0.202	1.28 (0.54–3.08)	0.577	16.9	0.127	1.72 (0.67–4.39)	0.259
**Chemotherapy**									
No	19	6.9		1		8.4		1	
Yes	31	18.2	0.017	0.39 (0.19–0.81)	0.012	24.2	<0.001	0.17 (0.07–0.38)	<0.001
**MRS+NLR**									
Low risk	20	26.23		1		38.50		1	
Moderate risk	8	12.70	<0.001	8.50 (2.90–24.90)	<0.001	12.70	0.002	7.58 (2.60–22.10)	<0.001
High risk	22	5.67	<0.001	57.04 (17.53–185.61)	<0.001	8.40	<0.001	12.12 (4.96–29.62)	<0.001

Supplementary Table S11. Subgroup analysis of DFS and OS according to combined MRS and NLR in patients with pCCA.

Variable	*n*	DFS (month)	Univariate	Multivariate		OS (month)	Univariate	Multivariate	
			p-value	HR (95%CI)	*p*-value		*p*-value	HR (95%CI)	*p*-value
**R status**									
R0	17	27.6		-		41.5		-	
R1	18	9.9	0.496	-	-	15.9	0.370	-	-
**Tumor morphology**									
ID mix	17	14.7		-		32.8		-	-
Without ID	18	15.9	0.745	-	-	16.6	0.237	-	-
**Differentiation**									
Well	26	12.9		-		25.9		-	
Moderate/poor	9	16.6	0.887	-	-	18	0.704	-	-
**N stage**									
N0	14	27.6		1		32.8		1	
N1	21	9.9	0.057	1.09 (0.38–3.11)	0.872	15.9	0.060	0.91 (0.30–2.78)	0.867
**Stage**									
0-II	8	27.6		1		NR		1	
III-IV	27	12.9	0.105	1.36 (0.35–5.19)	0.658	18	0.072	1.80 (0.37–8.79)	0.465
**Chemotherapy**									
No	12	12.7		-		13.6		1	
Yes	23	15.9	0.415	-	-	27	0.175	0.78 (0.32–1.93)	0.595
**MRS+NLR**									
Low risk	12	47.77		1		67.10		1	
Moderate risk	13	14.77	<0.001	3.98 (1.35–11.78)	0.012	16.70	0.005	5.13 (1.54–17.07)	0.008
High risk	10	8.00	<0.001	15.05 (4.00–56.64)	<0.001	8.80	<0.001	17.39 (4.03–75.01)	<0.001

Supplementary Table S12. Subgroup analysis of DFS and OS according to combined MRS and NLR in CCA patients receiving chemotherapy.

Variable	*N*	DFS (month)	Univariate *p*-value	Multivariate HR (95%CI)	Multivariate *p*-value	OS (month)	Univariate *p*-value	Multivariate HR (95%CI)	Multivariate *p*-value
**Tumor location**									
iCCA	31	18.2		-		24.2		-	
pCCA	23	15.9	0.446	-	-	27	0.443	-	-
**R status**									
R0	29	22.9		-		29		-	
R1	25	13.6	0.497	-	-	18.7	0.220	-	-
**Tumor morphology**									
ID mix	18	14.5		-		29		1	-
Without ID	36	18	0.542	-	-	19.9	0.132	1.62 (0.78-3.35)	0.195
**Differentiation**									
Well	44	18.2		-		26		-	
Moderate/poor	10	14.4	0.442	-	-	18	0.440	-	-
**N stage**									
N0	34	22.9		1		28.7		1	
N1	20	8.9	0.012	1.09 (0.38–3.11)	0.872	16.9	0.031	1.28 (0.64–2.54)	0.488
**Stage**									
0-II	16	25.9		-	-	29		-	
III-IV	38	13.6	0.360	-		22.1	0.299	-	-
**MRS+NLR**									
Low risk	23	41.5		1		58.9		1	
Moderate risk	15	13.6	<0.001	3.85 (1.68–8.61)	0.001	16.7	<0.001	5.10 (2.19–11.92)	<0.001
High risk	16	7.8	<0.001	21.20 (7.96–56.45)	<0.001	16.9	<0.001	8.83 (3.56–21.91)	<0.001

Supplementary Table S13. Subgroup analysis of DFS and OS according to combined MRS and NLR in CCA patients not receiving chemotherapy.

Variable	*N*	DFS (month)	Univariate	Multivariate		OS (month)	Univariate	Multivariate	
			p-value	HR (95%CI)	*p*-value		*p*-value	HR (95%CI)	*p*-value
**Tumor location**									
iCCA	19	6.9		-		8.4		1	
pCCA	12	12.7	0.249	-	-	13.6	0.185	0.23 (0.07-0.74)	0.013
**R status**									
R0	20	9.5		-		10.7		1	
R1	11	8.4	0.264	-	-	8.4	0.145	0.92 (0.37-2.31)	0.861
**Tumor morphology**									
ID mix	17	8.4		-		10.7		-	-
Without ID	14	9.0	0.133	-	-	9	0.488	-	-
**Differentiation**									
Well	23	9.5		-		26		-	
Moderate/poor	8	4.9	0.456	-	-	18	0.440	-	-
**N stage**									
N0	14	9.0		-		9		-	
N1	17	8.4	0.601	-	-	10.2	0.710	-	-
**Stage**									
0-II	16	3		-	-	70.4		1	
III-IV	38	28	0.043	-		9	0.035	8.74 (0.89-85.89)	0.063
**MRS+NLR**									
Low risk	9	47.8		1		67.1		1	
Moderate risk	6	12.7	0.041	2.95 (0.78–11.50)	0.118	13.6	0.094	5.51 (1.28–23.67)	0.022
High risk	16	4.1	<0.001	13.33 (3.39–52.40)	<0.001	5.2	<0.001	29.64 (6.42–136.89)	<0.001
